# Development of a national Distress Brief Intervention: a multi-agency service to provide connected, compassionate support for people in distress

**DOI:** 10.1186/s12913-025-12469-3

**Published:** 2025-04-04

**Authors:** Ambrose J. Melson, Karen Wetherall, Kevin O’Neill, Margaret Maxwell, Eileen Calveley, Martin McCoy, Rory C. O’Connor

**Affiliations:** 1 School of Health & Wellbeing, University of Glasgow, Glasgow, UK; 2 Distress Brief Intervention Central Team, NHS Lanarkshire, Wishaw, UK; 3 Centre for Healthcare and Community Research, University of Stirling, Stirling, UK; 4 University of the Highlands and Islands, Inverness, UK

**Keywords:** Distress Brief intervention (DBI), Complex intervention development

## Abstract

**Background:**

Mental health problems, self-harm and suicide are major public health concerns. Following national strategic commitments to improve the response and follow-up support for adults in Scotland presenting to frontline services in emotional distress, this study describes the development of the first national Distress Brief Intervention, a multi-agency service to provide connected, compassionate support for people in distress.

**Methods:**

The six step Intervention Mapping protocol was used to account for the complexity of the intervention and to guide development, testing and implementation. Data/information sources comprised: literature and evidence review; delivery partner and stakeholder consultations (*n* = 19); semi-structured interviews and/or focus-groups with frontline services staff experienced in responding to distress (*n* = 8); interviews and/or focus groups with adults with experience of distress (*n* = 9); feedback from test training for staff (*n* = 16); self-assessed confidence ratings provided by staff immediately before and following training (*n* = 388).

**Results:**

We developed a time-limited, two-level, complex intervention for adults experiencing emotional distress, provided by ‘frontline’ statutory services (primary and acute healthcare, police, ambulance) and third-sector community organisations in Scotland. Intervention components included competency-based training programmes for staff, information, protocols and guidance for providers, personalised distress management planning and behaviour change tools. During the development phase, 525 intervention providers (*n* = 472 frontline statutory service staff; *n* = 53 third-sector community organisation staff) completed training programmes in four pilot areas in Scotland. Training evaluations from 388 providers (74%) indicated significantly greater confidence following training on key competencies.

**Conclusions:**

A multi-agency national Distress Brief Intervention was systematically developed and implemented in a range of non-specialist frontline and community settings in Scotland. Up-take of training and evaluations of training indicate it is highly acceptable to potential providers and improves key competencies. Following independent evaluation, the Distress Brief Intervention has been rolled out nationally across the whole of Scotland, and has significant potential as a model of care and prevention internationally, including countries with low statutory health resources.

**Supplementary Information:**

The online version contains supplementary material available at 10.1186/s12913-025-12469-3.

## Introduction

Distress, defined as individuals’ self-reported mental health complaints and symptoms [1], is a widely experienced emotional state often resulting from a stress response to demanding or threatening experiences and which is characterised by difficulties coping, altered emotional state, discomfort and the expression of discomfort and harm [2–4]. Behavioural and psychophysiological responses can present as symptoms of common mental health problems including depressive symptoms, anxiety, aggression, irritability, fatigue, feelings of isolation, and substance misuse [5]. In response to distressing internal and external stressors (including but not limited to feeling trapped by painful thoughts and feelings or experiencing a breakdown in a relationship or financial precarity), some may also experience suicidal crisis and/or engage in self-harm, the immediate experience and consequences of which may further escalate one’s distress and contribute to a cycle of increasing risk [6, 7]. While distress can be more common among those with mental illness or disorder [8–11], distress is also a nonpathological emotional response to challenging social, relational and economic factors, which may be set against a backdrop of possible biological, psychological and psychiatric vulnerability and life events [6, 7, 12–14].

People in distress often present to primary and secondary healthcare services, where higher levels of distress are reported more in frequent rather than infrequent users [15]. Moreover, distress experienced as a result of co-occurring life challenges or stressors can lead to the involvement of other frontline and emergency services including ambulance and police services [16, 17]. Although statutory frontline services are often the first point of contact for individuals in distress and crisis [15, 18, 19], notwithstanding responses such as Psychological First Aid which are intended to mitigate risk of an acute stress response following a major incident or trauma event [20–22], care pathways and support may be limited or non-existent where there is an absence of physical or mental ill health diagnoses [23]. Indeed, to our knowledge there are no defined care pathways designed specifically for those who present to healthcare services in distress.

A lack of parity in the availability of services to treat mental versus physical health problems means frontline staff can feel ill-equipped and lack confidence to respond effectively to those in distress [24, 25]. In addition, shortcomings in service design and limited provision for those presenting in distress, mean the frontline response has tended to be inconsistent, with service users receiving suboptimal care that falls well short of their needs and may lead to repeat presentations across multiple services [16, 19, 25]. The social and economic burden associated with the disproportionate use of frontline services by those in distress is significant [26–28] and professionals report feelings of frustration, hopelessness and helplessness [24, 25].

### The role of the third sector in the delivery of mental health services

Third sector organisations (i.e., non-governmental and not-for-profit organisations, including charities and voluntary/community groups) are increasingly recognised for their critical and distinctive role in providing community-based physical and mental health support. Often grounded in recovery and person-centred paradigms, third sector service provision offers increased flexibility with staff and services to be able to work adaptively to tailor support to meet the needs of service users in the community [29–31]. Further distinctive features of third sector provision can include the delivery of services by workers with their own lived experience of mental health problems, helping to overcome traditional power imbalances between professionals and users based on a shared lived experience, providing a role model for individual recovery and engaging users with services and the community [32–34]. In these contexts, social rather than clinical models of mental health support are employed alongside strengths-based approaches [32]. While the third sector represents a significant opportunity for strengthening mental health service provision, there are often insufficient resources to sustain and develop services, low recognition of the breadth of support and expertise available as well as difficulties working collaboratively with statutory service partners to provide integrated and coordinated support [31, 32].

### The development of a Distress Brief Intervention in Scotland

Successive mental health and suicide prevention strategies in Scotland have recognised the significant unmet need surrounding distress [29, 35, 36]. To address this the Scottish Government proposed a multi-site pilot programme to facilitate the development, implementation and evaluation of a novel brief intervention for those in distress [37]. Its aim was to provide a service response for people in distress, who come into contact with non-specialist statutory health, social and law enforcement services and to offer time-limited community-based support following a frontline presentation, for example, to the emergency department [37]. The brief intervention should be developed to support a wide range of distress presentations, irrespective of the cause or contributing circumstances [20–22]. As no such intervention of this type existed anywhere internationally [38] the Scottish Government initiated plans to develop a new Distress Brief Intervention for use by frontline and community services. The University of Glasgow and partners were commissioned to work with key stakeholders to develop the Distress Brief Intervention. The present study aims to describe the systematic development of this first national Distress Brief Intervention (DBI).

## Methods and results

Given the iterative nature of our intervention development, which draws on a wide range of information sources and research activities, we have sought to minimise repetition and present study methods alongside the results of the development process consistent with other similar studies (e.g. [39]).

### Ethics approval and consent to participate

The development work was considered by the West of Scotland Research Ethics Service and designated a service development. Service development is distinct from research and exempt from ethical review following the UK Governance Arrangements for Research Ethics Committees. Written informed consent was obtained from all staff members and those with lived experience of distress and service users who contributed to interview and focus groups discussions as part of the development work. Consent included the use of anonymised quotes within training materials and reports.

### Intervention development and timeline

In the absence of bespoke interventions for responding to people in distress [38], we sought to develop the Distress Brief Intervention (DBI) guided by established frameworks such as a the Medical Research Council’s guidelines on the development of complex interventions [40, 41]. As the intervention would target those in distress across a range of settings, it needed to be flexible to allow for its delivery and use in response to a wide range of distress presentations by professionals across different service and organisational contexts. As we anticipated that many of those in distress may also have a history of suicidal thoughts, behaviours and self-harm, the development of the training components of the intervention was informed by the integrated motivational-volitional model of suicidal behaviour [6, 42] and the diathesis-stress model [43]. To account for its complexity and to provide an explicit description of key decisions and assumptions, intervention development was guided by the six steps described in the Intervention Mapping protocol [44, 45]. Intervention Mapping is a well-established method for complex intervention development, which has been used in health and community settings for a range of different intervention programme types and outcomes [39, 46–49].

**Fig. 1:**
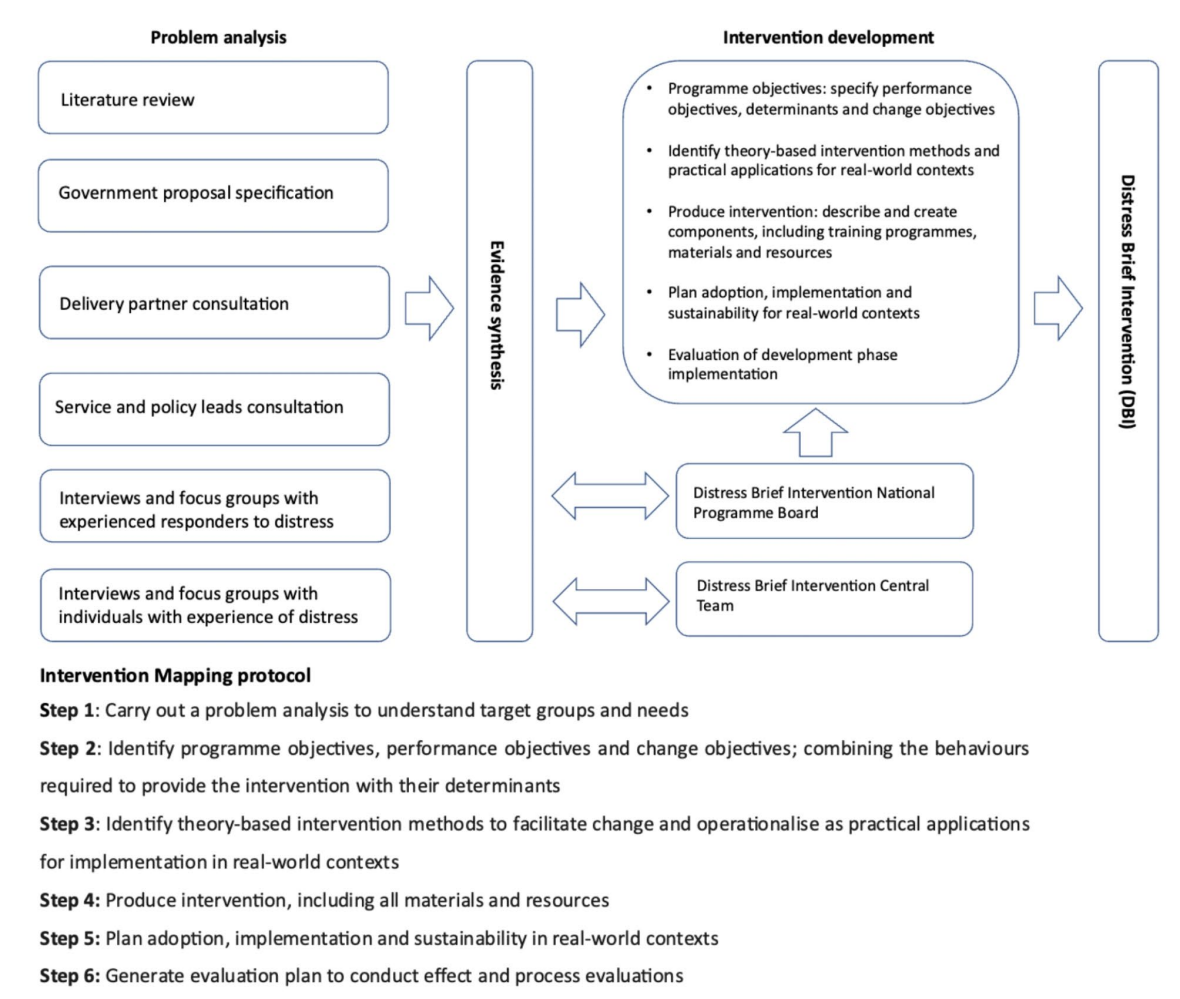
Overview of the problem analysis and intervention development process for the Distress Brief Intervention

The intervention development phase reported here took place over an 18-month period between September 2016 through to March 2018. This development phase included implementation in one pilot area from June 2017, followed by implementation in three other pilot areas by October 2017, and a further period of up-scaling through to March 2018. Given the relatively short period of time available to develop and implement the intervention, we took a pragmatic approach to the Intervention Mapping protocol steps. Figure 1 presents an overview of the problem analysis, the six steps of the Intervention Mapping protocol and the intervention development process for the DBI.

### Intervention Mapping Step 1: problem analysis

Our problem analysis at Step 1 of the Intervention Mapping protocol had two aims. The first aim was to understand the experience of those seeking help when in distress, as well as the response and support currently provided by frontline services. This began with a national engagement exercise, coordinated by the Scottish Government, which informed a preliminary specification for the DBI [37], and that was used as our starting point. A summary of key specifications can be found in Table 1 and these were explored through our second aim: to understand the needs of frontline and third sector services when responding to people in distress, including the acceptability of the proposed intervention components, training needs and the factors that would facilitate or impede implementation and adoption of the intervention in complex service settings.

**Table 1:** Summary of government specification for a Distress Brief Intervention [37]

∙ There should be a joint role for frontline statutory services and third sector community organisations in providing brief and time-limited support for individuals presenting to frontline services in distress.
∙ The intervention should be suitable for delivery by non-specialists across a wide range of frontline services and contexts and a staff training programme would, therefore, be needed to support delivery of the intervention.
∙ Frontline services will provide a ‘Level 1 response’ at point of presentation, which, in turn, may lead to an offer of referral to receive an additional ‘Level 2’ enhanced response based in the community.
∙ The Level 2 follow-up support should involve contact initiated within 24 hours and last up to 14 consecutive days.
∙ The intervention should be piloted in four parts of Scotland and subject to an independent evaluation

Table 2 describes the key sources of information and activities used in the problem analysis. We then present a summary of the evidence and synthesis of findings and decisions that we used to inform subsequent steps of the Intervention Mapping protocol. Activities took place in parallel, therefore, emergent findings informed subsequent activities.

**Table 2:** Main activities and sources of information used in the problem analysis

Activity Categories	Sources
**Literature review** Review of underpinning evidence, government specifications and relevant services or programmes.	∙ Scottish Government stakeholder engagement review findings and proposed specification for a Distress Brief Intervention ∙ Information Services Division review of evidence for short contact interventions ∙ Review of existing programmes and initiatives
**Distress Brief Intervention National Programme Board** Representatives of government, national agencies, programme partners and pilot sites providing strategic direction, coordination, planning as well as multisectoral oversight for key decisions.	∙ 3-hour monthly meetings (*n* = 15)
**Delivery partner and stakeholder consultation** Consultation with national agencies including Police Scotland, Scottish Ambulance Services, NHS 24; health services and boards, general practice, accident and emergency; third sector community services; government/policy leads; anti-stigma and discrimination organisations.	∙ Semi-structured interviews with pilot area leads (*n* = 5) ∙ Semi-structured interviews with national delivery agencies (*n* = 2) ∙ Site visits to pilot areas (*n* = 2) ∙ Structured discussion with policy leads, health boards and other stakeholders (*n* = 6) ∙ Local partnership development meetings (*n* = 4)
**Lived experience of distress** Views and experiences of individuals with experience of distress and use of services.	∙ Focus groups with third sector service users (*n* = 4) ∙ Semi-structured interviews with third sector service users (*n* = 4) ∙ Service users’ representatives round table structured discussion (*n* ~ 104 services users)
**Staff experienced in responding to distress** Views and experiences of frontline staff.	∙ Focus groups with Police Scotland staff (*n* = 3) ∙ Focus groups with third sector services staff (*n* = 3) ∙ Semi-structured interviews with GPs (*n* = 1) ∙ Semi-structured interviews with third sector services staff (*n* = 1)

### Interviews and focus groups with those with lived experience of distress and frontline services use

We carried out semi-structured interviews (*n* = 4; 3 female and 1 male participant) and focus groups (*n* = 4 groups; 11 female and 12 male participants) with a range of stakeholders and facilitated structured discussion activities at service user events (*n* = 1 event; 104 participants), to better understand the lived experience of people in distress and their experiences of frontline services, and what they hoped a DBI would provide. Due to the potentially sensitive nature of discussions, we offered those interested the choice of participating in an interview or a focus group. Interviews and focus groups were facilitated by AJM, KW and EC, with interviews lasting between 38 and 50 min and focus groups between 55 and 83 min. Recordings were transcribed, coded and thematically analysed to yield interrelated ideas and themes guided by step-specific and more general study aims. Their insights highlighted several important themes, as summarised below. Further information from the interviews and focus groups, including topics covered and additional quotes, can be found in Additional File 1.

#### Experiences of service use and seeking help by those in distress

Those with experience of distress who had contact with frontline services reported mixed experiences of their encounters and how they were treated by frontline services. Some highlighted that their encounters were helpful and they felt listened to but others reported less positive experiences. Several interviewees described the contrast in interactions with frontline services, with some staff clearly signalling their interest and care for the person while others focused on tasks:

*“You can tell by their body language and the tone in their voice*,* and the way they speak to you*,* the words that they use. It’s sort of*,* you know*,* are they there to actually have a conversation with you*,* or are they just there to tick a box? And you get that straight with them*,* the minute they walk in the door”* [Person with experience of distress and contact with frontline services; interview].

#### Parity of esteem between mental and physical health

Those with lived experience of distress and contact with frontline services did not always feel their distress was treated on a par with physical health. The perceived lack of parity was particularly salient for those who presented to frontline services with suicidal thoughts or self-harm. Some participants described experiencing stigmatising attitudes among frontline staff, who considered them to be attention seeking:

*“…but it all goes back down to the listening*,* because I mean*,* you can get some people*,* especially up at A&E*,* they’re just like ugh*,* kind of you’re an annoyance*,* really. I’ve been in A&E a couple of times from overdosing when I’ve been quite distressed*,* and I’ve had some quite negative reactions from staff that are just kind of like*,* well*,* you’re just attention seeking*,* sort of attitude.”* [Person with experience of distress and contact with frontline services; interview].

When describing experiences of follow-up support and service response a common theme was the limited support available following initial treatment and assessment. This included feeling dismissed as not requiring further support and that formal follow-up support or referral to other services was rarely offered. Instead, those with experience of distress were encouraged to utilise family member support or to contact their GP.

#### Views on a Distress Brief Intervention

Those with experience of distress and contact with frontline services were asked about the type of follow-up support that might be helpful as well as their views on the proposed DBI (see Table 1). Overall, those with lived experience felt a DBI was desirable and would be acceptable, though there were clear caveats, including the scale of the perceived need and whether the DBI would be able to cope with demand:

*“I think this looks like a great idea but you are going to be slammed in your pilot. And I hope that you’re prepared to be completely overwhelmed*,* because you will be for the exact reason that this person’s just shared*,* because there’s just a big black hole where there should be this service.”* [Person with experience of distress and contact with frontline services; focus group].

Adding to concerns about the potential scale of demand, some individuals felt the DBI would be used by existing frontline and community mental health services as a ‘dumping ground’ for individuals whose needs are not met through existing care pathways. Views also varied on whether the proposed 14 consecutive days of follow-up support available through the DBI would be sufficient. Some recognised that this was potentially a more intensive, time-limited, service, while others felt the time available would not be sufficient to develop trust as a basis for meaningful progress. Potential impacts included providing a listening ear at a critical time, which might prevent a future crisis and possibly suicidal behaviour.

Those with lived experience of distress suggested several facilitators to the success of the DBI, including involvement of people with lived experience of distress within training programmes for staff. Engaging with the same practitioner over the course of the period of support was also seen as helpful by those with experience of distress, providing the opportunity to build rapport and an enhanced sensitivity to the person’s current distress and needs, as well as reducing the requirement to cover old ground and retell their story. Additional suggestions that could enhance the acceptability and benefits of follow-up support included the importance of knowing when contact will be made and having the option of individual face-to-face support and access to information and self-help groups or activities that can help to manage and prevent future distress.

### Interviews and focus groups with staff experienced in responding to distress

We carried out six focus groups (*n* = 3 Police Scotland groups; 2 female and 18 male participants; *n* = 3 third sector mental health support worker groups; 10 female and 4 male participants) and two semi-structured interviews (*n* = 1 female third sector mental health support worker; *n* = 1 female General Practitioner) with staff who regularly respond to those in distress. We again sought to accommodate the preferences of those participating by offering interviews or focus groups, with most organisations and services preferring the logistics of releasing groups of staff for focus groups over individual interviews. Staff interviews and focus group were facilitated by AJM, KW and EC, with interviews lasting between 16 and 35 min and focus groups between 60 and 99 min. Audio recordings were transcribed or summarised prior to coding, categorisation of related codes and the identification of themes. In these discussions we sought to understand the existing response to distress and elicit staff members’ views on the proposed DBI, including its fit with their current role and any barriers and facilitators to implementation. The views of staff follow, with the views of third sector staff preceding those of Police Scotland within each theme. Further information from the interviews and focus groups is provided in Additional File 1.

#### Potential benefits of the Distress Brief Intervention

Third sector mental health support workers highlighted the potential for the DBI to prevent escalation of distress to more serious and resource-intensive care. The plans to offer up to 14 days of support in the community were seen as preferable to the short time that the person may spend with a frontline services worker when in crisis with few options for ongoing support. Providing an initial contact within 24 hours was also seen as an important feature of the proposed brief intervention, offering hope and the prospect of support in the near term:

*“It could be really preventative in the fact that you go to hospital*,* you wait the six hours and sometimes you’ve reached rock bottom and you want somebody to say ‘I hear what you’re saying*,* that must be really*,* really hard for you*,* here’s what I can do for you’ which is very limited but if you can say ‘a worker’s going to contact you in 24 hours to see how and if they can help you’ that’s something for that person to hang onto*,* so in terms of risk management*,* it’s helping them because they’ll hang on because they know that’s coming.”* [Third sector mental health support worker; focus group].

Third sector mental health support workers also viewed the DBI as empowering and enabling, providing a time-limited supportive framework at the time of acute need that could then serve as the basis for future self-management. By listening to individual needs, signposting the person to available services, and supporting them in accessing these for the first time, a journey towards recovery could be facilitated.

Police Scotland staff suggested the DBI would have a range of benefits for people in distress, including providing someone to talk to and offer advice, as well as more specialist community support soon after the immediate crisis. Perceived benefits to Police Scotland and its staff included potential time-savings and reductions in repeated presentations or attendances. Additionally, Police Scotland staff emphasised that being able to leave the person in distress with the offer of further contact within 24 hours would mean that the presentation or attendance ended on a positive note:

*“…every little wee bit that can help us*,* that we can say to somebody… before we were just walking out of somebody’s house*,* while not having brought it to a total conclusion*,* we can now say*,* right*,* here’s a card*,* you’re going to be getting a phone call in 24 hours…”* [Police Scotland officer; focus group].

#### The interplay between the Distress Brief Intervention and one’s current role

Generally, the DBI was felt to fit comfortably within the third sector community mental health support worker’s role and expectations for an appropriate intervention for those in distress. This included the opportunity to listen and establish a relationship with a vulnerable person that would enable workers to work alongside the person to explore the reasons for their distress, how it could be prevented or managed in the future, as well as signposting towards appropriate services.

Police Scotland officers viewed dealing with people in distress as part of their duty of care, including safeguarding the person and helping them to access available services. During our discussions with officers, distress was often conflated with suicide risk, that this constituted a medical or clinical concern and should be assessed by those with clinical expertise. Where distress was related to suicide risk, there was a perception that the DBI might have limited impact upon the response of Police Scotland officers as they would still feel the need to seek clinical assessment. On the other hand, some officers felt that the DBI might engender confidence in a decision not to seek clinical assessment, but this would depend on the existence of clear protocols to instruct them and to which they could adhere:

*“So anything that you can do*,* for instance*,* the protocol is the crisis team or Breathing Space speak to that person and then speak to the cops and say yeah*,* they’re okay*,* they’re a bit low. They’ve agreed to meet us tomorrow or… I’m happy that that is the case. It gets documented down*,* the cops go. It still might take a bit of time but it won’t take four hours.”* [Police Scotland officer; focus group].

#### Potential challenges/barriers to the implementation of the Distress Brief Intervention

Among third sector community mental health support workers there was a perception that frontline services had a low awareness of the support that could be offered by the third sector to people experiencing distress. The third sector workers also perceived a disparity in professional status, noting that their professionalism and skills were not always recognised by frontline service workers, and that this may jeopardise a brief intervention service which depended on smooth and efficient inter-agency working. As described by one worker, poor inter-agency communication was seen as part of the problem:

*“no-one can share anything with anyone*,* really*,* and so you’ve got the police that are running around*,* you’ve got third sector*,* like voluntary organisations trying to support*,* not knowing what’s going on*,* you’ve got psychiatry*,* and no-one’s speaking to each other*,* and you’ve got a person that can go from pillar to post and back and forth and round and round…”*[Third sector mental health support worker; focus group].

While the third sector community mental health support workers felt the brief time-limited nature of the intervention would be appropriate for many people in distress, some anticipated challenges bringing the support to an end. To some extent it was felt this could be mitigated by managing expectations of those receiving support and ensuring post-support plans were in place. The careful management of the initial implementation of the DBI was considered vital by third sector workers, as rapid growth and scale would prove challenging for a new service and may add to strain felt across the sector if the DBI referred their service users onwards to other established services.

Challenges or barriers identified by Police Scotland officers also included concerns about capacity and the potential for the DBI to add to police responsibilities and workload. For example, officers queried how many attempts would be made by the third sector workers to contact the person in distress following a referral and whether unsuccessful contacts would be referred back to the police. Further challenges highlighted by Police Scotland officers included the value and credibility of training. Alongside a view that they were expected to undertake too much training, the credibility or expertise of those delivering mental health-related training within Police Scotland was questioned by some officers.

#### Potential facilitators to the implementation of the Distress Brief Intervention

The third sector workers cited several facilitators to the implementation of the DBI. Some of these provided recommendations for the type of support that should be available, for example, activities that would help the person in distress to access and engage with other services beyond the 14 days of support available.

Other facilitators highlighted by third sector workers included the provision of training for frontline statutory service workers that ensures they have a clear understanding of the third sector organisation’s role and remit within the DBI. It was believed this would contribute to effective inter-agency working between third sector and statutory frontline services. Recognising the importance of initiating follow-up contact within 24 hours of the referral being made, and that repeated contact attempts could place a strain on resources, the third sector workers also recommended that an explicit protocol should guide efforts to establish follow-up contact.

*“It’s about confidence in what the remit is and what we do and what we don’t do*,* and about clarity”* [Third sector mental health support worker; focus group].

### Consultations with delivery partners, service and policy leads and members of the Distress Brief Intervention National Programme Board

Throughout the development phase we carried out consultations with representatives of delivery partners and services, policy area leads and members of the Distress Brief Intervention National Programme Board. These consultations provided strategic perspectives on the intervention development, workforce needs and expectations within and across organisations, as well as views on the potential components and features of the DBI. Audio recordings and notes of interviews were combined with minutes of meetings to identify key issues, summarised as concerns and recommendations in Table 3.

**Table 3:** Summary of stakeholder (delivery partner, service and policy leads, Distress Brief Intervention National Programme Board member) views on the Distress Brief Intervention

Intervention feature	Summary of concerns and recommendations
**Clear oversight and central planning**	∙ A clear programme structure, with central coordinating body, would ensure consistency and communication across agencies and pilot sites. ∙ A board constituting expertise and representation of partners and stakeholders should provide oversight and be consulted on key decisions.
**Two-level**,** frontline and community-based**,** response and support**	∙ The Distress Brief Intervention specification described a two-level response, with frontline providers providing a ‘Level 1 response’ at point of presentation, which in turn could lead to an offer of referral to receive an additional ‘Level 2’ enhanced response based in the community. ∙ There was consensus among stakeholders that both frontline and community-based responses were needed to address the range of distress presentations and intensity and type of support needed. ∙ Some stakeholders emphasised that the Level 1 response, provided at point of presentation, would be constrained by time pressures and it would not be possible to provide a standalone intervention in the frontline setting. ∙ There was uncertainty about whether some staff providing a Level 1 response staff would feel competent to determine the need for an enhanced Level 2 response, which may become a barrier to staff engaging with the intervention.
**Intervention duration and speed of response**	∙ While a Level 1 response was anticipated to be relatively brief, stakeholders were unsure what the optimal duration should be for the enhanced Level 2 response provided in the community. In the absence of comparable interventions for distress, most stakeholders felt support lasting up to 14 consecutive days was appropriate for initial piloting and evaluation. ∙ For those referred to enhanced community-based Level 2 response, stakeholders felt a 24-hour target for initiating follow-up contact would offer quick and efficient transfer from statutory to community services for follow-up support. Notably, several organisational or service leads emphasised that this speed of follow-up contact would be a vital condition for Level 1 frontline staff engaging with the intervention.
**Target population and eligibility**	∙ Clear and simple eligibility criteria and operational definitions of distress were viewed as necessary for consistency and utility across different non-specialising frontline services and contexts. ∙ The operational definition of distress should seek to be inclusive of a wide range of presentations, as well as de-medicalise and normalise distress as a response to stressful social, relational, and economic events. ∙ The process of determining eligibility and need for the intervention should not amount to an assessment of risk.
**Intervention delivery partners**	∙ There was broad agreement among stakeholders that consistency across sectors and settings was needed to support people in distress, with consensus that a core set of frontline services should provide an initial Level 1 intervention response. ∙ There also broad support for the Level 2 community-based response to be met by established third sector community mental health support services. This should be the standard model of providers in each of the four pilot areas.
**Critical role of compassion**	∙ To address the needs of those in distress there was agreement that the intervention should be underpinned by a compassionate approach to responding to distress. ∙ Some stakeholders considered that a compassionate response required time and resources that already stretched frontline services were not well placed to provide. Other concerns raised by stakeholders included whether a compassionate approach would be readily adopted by those frontline services where compassion is not an established feature of practice.
**Referral process**	∙ The referral process linking the Level 1 frontline service response to the Level 2 community response was a key concern for frontline service leads. A process that is time-consuming, inefficient, or required non-standard systems or actions was felt to be a potential barrier to uptake and use of the intervention. ∙ The secure and timely transfer of information from the Level 1 referrer to the Level 2 community service, with confirmation of receipt, was also a concern of frontline services leads. This process was viewed as an important facilitator, with frontline service staff engagement with the intervention likely to depend on their confidence that immediate follow-up support would be available in-line with expectations. ∙ Of importance to third sector service leads was establishing a referral process that facilitated high quality, consistent and relevant information. Based on past experiences providing follow-on support for frontline services, poor quality referral information and systems were a barrier to the community support responding efficiently and appropriately to referrals.

### Problem analysis: synthesis and decisions

The problem analysis and the integration of the perspectives of a range of different stakeholders led us to identify critical issues to consider during further development of the DBI. A range of proposals were considered by the Distress Brief Intervention National Programme Board and then taken forward in the subsequent steps of the Intervention Mapping protocol and development.

#### Need and acceptability of Distress Brief Intervention

The problem analysis confirmed that the current statutory service response to distress did not meet the needs of those presenting in distress nor enable frontline services staff to respond effectively. While staff working in frontline and third sector support sectors are experienced in responding to distress, frontline services staff are overburdened, with limited capacity to provide additional time-intensive interventions and administrative tasks. Existing options for onward support or referral for those in distress are limited. DBI– presented as an enhanced response to distress at point of initial presentation (Level 1), followed by the option of further community-based support, initiated within 24 hours, and lasting up to 14 consecutive days (Level 2)– was seen as a valuable and broadly acceptable intervention by those with lived experience of distress, frontline and community services and other key stakeholders.

#### Target population and definition of distress

Suitable eligibility criteria, including an operational definition of distress, which could be applied consistently by a wide range of non-specialist frontline services and contexts were sought. The novelty of the intervention, its status as a pilot, and need for broad applicability across different services and types of distress presentation led to a consensus that the target population should be adults aged 18 years and over in distress. Distress was subsequently operationalised as “*an emotional pain for which the person sought*,* or was referred for*,* help and which does not require (further) emergency service response”.*

#### Providers of the Distress Brief Intervention Level 1 response

A multi-agency frontline response was considered necessary to ensure the DBI was able to address the range of needs of different population subgroupings, which, in principle, would also lead to a more equitable burden associated with providing the intervention. Stakeholders also believed that the DBI should support a standardised and consistent response to distress, regardless of where or when the person in distress presented for help. A core set of frontline services therefore emerged as preferred providers of the Level 1 intervention response provided at point of initial presentation. These were emergency department, primary care (general practice including out of hours), ambulance and police services.

There was consensus that established third sector community mental health support organisations should provide the follow-up Level 2 response in each of four pilot areas, based on their ability to offer person-centred support and management within the community. This was important to those with experience of distress who viewed busy frontline settings as being poorly suited to managing or easing their distress.

#### Minimising burden on frontline services

A recurring view among stakeholders was the limited time available to frontline services to respond to people presenting in distress, with some also expressing uncertainty or a lack of confidence in their ability to assess distress and determine whether, following a Level 1 response, the individual should additionally be offered an enhanced Level 2 response. Two crucial decisions followed these findings. These were firstly that the Level 1 response be closely aligned and embedded, as far as possible, within existing practical and administrative procedures of partner organisations. Secondly, the initial expectation that the Level 1 response should serve as a sufficient intervention for many individuals in distress was revised. A more modest set of expectations for the Level 1 response were proposed and it was decided that all those meeting the eligibility criteria for the initial DBI Level 1 response should automatically be offered a referral to receive the DBI Level 2 response.

#### Referrals process

The referral process linking the frontline services Level 1 response and community service Level 2 response should be simple, efficient, and part of or as similar to existing processes as possible, to avoid duplication of time and resources. The referral should be communicated electronically and securely, with confirmation of receipt. A process which incorporates these features was viewed as a prerequisite for the Level 2 service to be able to initiate contact with the person in distress inside 24 hours. Confidence in this process was also crucial to the acceptability and credibility of the DBI among those with experience of distress and the frontline services. Training should therefore be provided to support a reliable and effective referrals process as well as to minimise inappropriate referrals that do not meet eligibility criteria. The need to develop and establish a reliable referral process across different organisations and settings also played a role in decisions to limit the number of frontline services involved during the development phase.

#### Training needs

Although frontline services have significant practical experience responding to people in distress, confidence was mixed, with some services expressing concerns over a lack of training and that distress and decisions around safety and risk are more appropriately handled by those with clinical or specialist mental health training. Evidence from the problem analysis pointed towards mixed attitudes to mental health issues and distress and in some cases frontline services are lacking important knowledge and skills needed to respond effectively to those in distress. The views of those with experience of distress, the government’s stakeholder engagement exercise, and community services emphasised the need for a more consistent compassionate response to distress at all levels of intervention.

Given the complexity of the intervention and implementation contexts, a training programme would be needed to support delivery of the intervention. This would need to address knowledge and understanding surrounding distress, include coverage of self-harm and suicide risk, attitudes to those in distress and provide the skills to deliver the intervention. Supporting materials would be needed and would form part of the training materials. A core theme of all training would be to establish a compassion-focused and trauma-informed response to distress. The different responses expected of the frontline statutory services and the third sector community support organisations, mean that separate training programmes for each level would be needed. To support a consistent response to distress across frontline services, the core training would need to be standardised across providers.

#### Oversight and central planning

Although local implementations were expected to reflect pilot area contextual factors, it was determined at an any early stage of the problem analysis that central planning and co-ordination of the intervention programme was crucial. It was proposed that a dedicated Distress Brief Intervention Central Team be formed, comprising programme manager, analytical and administrative roles. A Distress Brief Intervention National Programme Board comprising government policy area leads, delivery partners and stakeholders should be consulted on key decisions.

### Intervention Mapping Step 2: programme objectives

In this step, we designed the foundations of the intervention by specifying who and what will change as a result of the intervention. Work undertaken as part of this step began from the establishment of relevant programme outcomes, with these further described as performance objectives representing the basic actions or behaviours required of actors to achieve each outcome. Modifiable determinants of performance objectives were identified and used to generate change objectives which are specific changes in determinants needed to achieve performance objectives.

Decisions from the earlier problem analysis fed into an evaluability assessment [50], which identified thirteen programme outcomes for the DBI and which are outlined in a Theory of Change (Additional File 2, Figure S1). Three of these programme outcomes are the focus of the current development work:

- DBI Level 1 frontline staff in A&E, police and ambulance services, primary care or social work and other first responders who have undergone DBI Level 1 training have the skills, competencies and confidence to deliver a Level 1 intervention.
- DBI Level 2 practitioners have the skills and competencies to deliver a Level 2 intervention.
- People who receive a DBI Level 2 intervention feel less distressed and more able to manage future episodes of distress.

#### Programme outcomes for frontline and third sector workers to provide the Distress Brief Intervention

Two of the three programme outcomes were directly linked to training DBI providers. Given the different roles, settings and contexts of the staff providing the DBI Level 1 and Level 2 intervention responses, separate programme outcomes were identified for each. The performance objectives necessary to deliver programme outcomes were defined as the competencies to deliver the DBI. For each performance objective we sought to identify its key determinants, with our decision-making guided by findings from the interviews, focus groups and consultations conducted with individuals with experience of distress and experienced staff, reviews of existing staff training programmes, including brief intervention initiatives and mapping to established competency frameworks (e.g., [51–54]). From this analysis we identified key determinants of each performance objective as knowledge, attitudes and skills and generated specific change objectives for each. For example, in order that DBI Level 1 workers successfully ‘make an offer and execute a referral to Distress Brief Intervention Level 2’ (performance objective/ competency statement 3.5) it was necessary for Level 1 workers to have requisite knowledge and skills (determinants) to explain the purpose, nature and potential benefits of making a referral to the intervention as well as understand the process and actions involved.

Tables 4 and 5 provide examples of selected performance objectives/ competency statements and the specific change objectives for the two programme outcomes which addressed training. The full set of performance objectives/competency statements, determinants and change objectives for the training programmes were considered by the Distress Brief Intervention National Programme Board and can be found in Additional File 3 (Tables S3 and S4).

**Table 4:** Examples of selected performance objectives/ competency statements, determinants and change objectives for programme outcome: Distress Brief Intervention Level 1 frontline staff have the skills, competencies and confidence to deliver a Level 1 intervention

Performance objective / competency statement	Determinants	Change objectives
3.2 Provide relevant information and advice about mental wellbeing and distress	Knowledge & Skills	3.2 Providing relevant information Gives accurate information and guidance clearly and in a way which is appropriate to the individual
3.3 Identify and problem solve when faced with barriers to delivering Distress Brief Intervention	Skills	3.3 Barriers and concerns Identify and resolve possible challenges to delivering Distress Brief Intervention
3.4 Provide clear and accurate signposting information	Knowledge Skills	3.4 Signposting to other services or supports Knowledge of appropriate supports and services Clear communication and explanation of services and supports in a way which is appropriate to the person in distress
3.5 Make an offer and execute a referral to Distress Brief Intervention Level 2	Knowledge & Skills Knowledge	3.5 Referrals Able to explain the purpose, nature and value of referral to Distress Brief Intervention Level 2 for the person in distress Understand how to make a referral to Distress Brief Intervention Level 2

**Table 5:** Examples of selected performance objectives/ competency statements, determinants and change objectives for programme outcome: Distress Brief Intervention Level 2 practitioners have the skills and competencies to deliver a Level 2 intervention

Performance objectives / competency statements	Determinant	Change objectives
5.2 Provide person-centred support across all relevant aspects of the Distress Brief Intervention, working *collaboratively* with the person referred following distress throughout	Knowledge & Attitudes Knowledge & Skills Skills Knowledge & Skills	5.1, 5.2 Person-centred support Recognises and acknowledges the underlying determinants of distress in the context of personal histories, including trauma awareness Identifies proximal triggers or causes of distress Develops and records a support plan Provides signposting and information depending on the needs of the person referred following distress
5.3 Understand the importance and how to enact a compassionate response for distress	Knowledge & Skills Knowledge & Skills	5.3 Compassionate response Understands the importance, key skills and attributes of compassionate response Understands how to use compassion skills and attributes in the context of an initial contact and 14-day support
5.4 Use motivational interviewing techniques during Distress Brief Intervention contacts	Knowledge Skills Skills Skills Skills	5.4 Motivational interviewing Understands and acknowledges the ambivalence that the person referred following distress may feel about their actions and behaviour Uses basic motivational interviewing skills (e.g. OARS) to support Distress Brief Intervention interactions Is able to adapt to and roll with resistance during Distress Brief Intervention contacts Provides regular feedback and summaries to structure Distress Brief Intervention contacts and transition to other components of the Distress Brief Intervention Encourages personal responsibility
5.5 Use cognitive behavioural techniques to understand distress and identify suitable strategies of support	Knowledge Skills Skills	5.5 Cognitive behavioural approach Understands the relevance of a cognitive behavioural approach to distress Support a person referred following distress to explore interrelated thoughts and behaviours relevant to their distress Uses appropriate materials or tools to support a person referred following distress to understand and address unhelpful (i) thoughts, (ii) behaviours
5.6 Understands the relevance of health behaviour change and can support individuals referred following distress to change behaviours	Knowledge & Skills Skills Skills	5.6 Behaviour change methods and techniques Identify relevant methods and techniques of behaviour change to support the objectives of the individual referred following distress Adapt and communicate behaviour change strategies to the needs of the individual referred following distress Review and assess progress throughout

#### Programme outcome for people who receive a Distress Brief Intervention Level 2 intervention

To achieve the third programme outcome, ‘people who receive a DBI Level 2 intervention feel less distressed and more able to manage future episodes of distress’, a person in distress should engage with and receive a service that executes the performance objectives/ competencies underpinning the DBI Level 1 and Level 2 responses. An overview of a person in distress’s anticipated contact and involvement with the intervention, from initial presentation to exit, is outlined below alongside corresponding performance objectives/ competencies (descriptions of performance objectives can be found in Additional File 3 Tables S3 and S4).

- *Presentation and assessment*: Person in distress presents to/is attended by DBI Level 1 service and is assessed as eligible based on explicit criteria (performance objectives/ competencies 1.1, 1.2, 1.3, 2.1, 2.2, 2.3, 3.1).
- *Referral*: Person in distress is offered and receives a referral for further follow-up support provided by the DBI Level 2 service in the community (performance objectives/ competencies 3.2, 3.3, 3.4, 3.5).
- *Initial contact*: Person in distress receives contact from DBI Level 2 service within 24 hours and is offered support lasting up to 14 consecutive days (performance objectives/ competencies 4.1, 4.2).
- *Engagement and support*: Person in distress engages with the DBI Level 2 service and receives person-centred support lasting up to 14 consecutive days (performance objectives/ competencies 5.1, 5.2, 5.3, 5.4, 5.5, 5.6).
- *Exit*: Person in distress leaves DBI Level 2 service feeling less distressed and with further support, information, and personalised plans in place to manage their distress in future (performance objectives/ competencies 5.7, 5.8).

### Intervention Mapping Step 3: theory-based intervention methods and practical applications for real-world contexts

In Step 3, the key intervention components were identified. We selected theoretical methods optimally suited to bringing about the desired change in determinants of the performance objective using taxonomies of behaviour change methods (e.g., [55–57]), empirical evidence and stakeholder consultations. For each method we further identified practical applications, providing the basis of the DBI Level 1 and Level 2 staff training programmes and the intervention components leading people to feel less distressed. Tables 6 and 7 provide examples of selected change objectives, the linked methods of change and practical applications for inclusion within the DBI Level 1 and Level 2 training programmes.

**Table 6:** Examples of change objectives, theoretical methods and practical applications for the programme outcome: Distress Brief Intervention Level 1 frontline staff have the skills, competencies and confidence to deliver a Level 1 intervention

Change objectives	Theoretical methods	Practical applications
Understands that different feelings or attitudes about distress might influence their approach and practice to Distress Brief Interventions	Prompt reflection of past behaviour Credible source Provide information about health and emotional consequences	Reflective activity: encourage reflection of personal attitudes and their impact on recent experiences responding to distress. Record in journal or discuss Provide evidence-based information describing the impact of negative staff attitudes on the experience and outcomes of those in distress
Recognise that distress may present in various forms, including medically unexplained symptoms	Credible source Provide information about behaviour-health link Provide information about health and emotional consequences	Present evidence-based information and different examples of distress presentations Present written quotes from individuals with lived experience of distress
Awareness of important influences on distress (e.g. individual, social, cultural and environment) including stigma	Credible source Provide information about behaviour-health link	Present evidence-based information, and varied examples of potential influences on distress including health inequalities, social determinants, stigmatising attitudes, trauma experiences Provide theoretical framework for understanding interplay of factors that contribute to distress e.g. stress-vulnerability diathesis
Understands the value, and skills involved in an empathic frontline assessment of a person in distress Understands how to undertake empathic assessment in context of a frontline response to distress	Credible source Provide information about health and emotional consequences Instruction on how to perform a behaviour	Provide evidence-based information demonstrating the importance of interactions conducted sensitively and with empathy to those in distress, for example during psychosocial assessments Activity: encourage consideration of potential benefits of adopting empathic approach for: (i) a person in distress, (ii) frontline service staff responding to the person. Record in journal or discuss Provide guidance and examples of interpersonal communication and behaviour that facilitate clear and sensitive communication, for example: acknowledging and validating; paying attention; open questioning; body language and nonverbal behaviour; checking understanding; sensitive probing Handout/Aide memoire: provide concise evidence-based information, guidance and tips to support frontline staff to identify and explore distress presentations including self-harm
Understands the importance, key skills and attributes of a compassionate frontline response Understands how to use compassion skills and attributes in the context of a frontline response to distress	Credible source Provide instruction Provide information about health and emotional consequences Problem solving Reduce negative emotions	Provide information defining compassion and clarifying the core attributes and skills involved in providing a compassionate response to distress Reinforce importance of compassionate response using quotes from individuals with lived experience of distress Provide evidence-based information on the beneficial outcomes of responding with compassion for those in distress and frontline services workers Show video content, featuring those with lived experience of distress reporting negative experiences of frontline service conversations and offering advice on how future experiences can be improved Reflective activity: encourage reflection on frontline service worker’s experience responding to distress, with and without with compassion, and the reasons for this Provide information on several challenges or obstacles to responding with compassion: compassion fatigue, burnout, vicarious traumatic stress. Encourage practices consistent with self-compassion: self-kindness, common humanity, mindfulness
Able to explain the purpose, nature and value of referral to the Distress Brief Intervention Level 2 for the person in distress Understand how to make a referral to the Distress Brief Intervention Level 2 service	Credible source Provide information about behaviour-health link Instruction on how to perform a behaviour	Provide written information and advice on introducing and explaining the referral and potential benefits of accessing the Distress Brief Intervention Level 2 service Provide written information on seeking consent; accessing the referral form; recording accurate and informative referral information; sending the referral form to the Distress Brief Intervention Level 2 service according to the agreed protocol Provide example referral form, with annotation and tips provided Provide written information on steps following referral so that these can be communicated to the person in distress Handout/Aide memoir: provide summary of key steps and prompts for offering and executing the referral to the Distress Brief Intervention Level 2 service.

**Table 7:** Examples of change objectives, theoretical methods and practical applications for programme outcome: Distress Brief Intervention Level 2 practitioners have the skills and competencies to deliver a Level 2 intervention

Change objectives	Theoretical methods	Practical applications
Understand how to respond appropriately to a referral within 24 hours	Instruction on how to perform a behaviour Problem solving	Provide and discuss Standard Operating Procedures booklet, including ‘what if’ scenarios
Understands and acknowledges the ambivalence that the person referred following distress may feel about their actions and behaviour Uses basic motivational interviewing skills (e.g. OARS) to support Distress Brief Intervention interactions Is able to adapt to and roll with resistance during Distress Brief Intervention contacts Provides regular feedback and summaries to structure Distress Brief Intervention contacts and transition to other components of the Distress Brief Intervention Encourages personal responsibility	Provide information about behaviour-health link Provide information about health and emotional consequences Instruction on how to perform a behaviour Demonstration of the behaviour Behavioural practice/rehearsal	60 min e-learning module ‘Foundations of Motivational Interviewing’ Facilitator-led introduction to motivation and motivational interviewing techniques, as well as Distress Brief Intervention Toolkit resources: Decisional Balance, Importance and Confidence Rulers, Aide Memoir Role play activity: small groups engage in scripted role play of an interaction between the person in distress and practitioner and identify use of motivational interviewing techniques: Open questions, Affirmations, Reflective listening Summaries Practice activity: encourage weighing pros and cons, as well as importance and confidence, of a change in personal behaviour using Decisional Balance and Importance and Confidence Rulers Toolkit resources.
Understands the relevance of a cognitive behavioural approach to distress Support a person referred following distress to explore interrelated thoughts and behaviours relevant to their distress Uses appropriate materials or tools to support a person referred following distress to understand and address unhelpful (i) thoughts, (ii) behaviours	Provide information about behaviour-health link Provide information about health and emotional consequences Instruction on how to perform a behaviour Demonstration of the behaviour Behavioural practice/rehearsal	Facilitator-led introduction to cognitive behavioural approaches and Distress Brief Intervention Toolkit resources: What are my triggers and situations?; Problem-solving and Action Planning; Goal Setting; How is my mood at the moment?; Self-monitoring; If-Then Coping Plans Role play activity: small groups roleplay a scenario between a person in distress and practitioner and practice skills and techniques, including toolkit resources.
Identify relevant methods and techniques of behaviour change to support the objectives of the individual referred following distress Adapt and communicate behaviour change strategies to the needs of the individual referred following distress Review and assess progress throughout	Provide information about behaviour-health link Provide information about health and emotional consequences Instruction on how to perform a behaviour Demonstration of the behaviour Behavioural practice/rehearsal	Facilitator-led introduction to behaviour change and maintenance principles and techniques, with Distress Brief Intervention Toolkit resources: Problem-solving and Action Planning; Goal Setting; Self-monitoring; If-Then Coping Plans Role play activity: small groups roleplay a scenario between a person in distress and practitioner and practice skills and techniques, including toolkit resources.

The theoretical methods of change (and practical applications) that address the third programme outcome, namely that people who receive a DBI Level 2 intervention feel less distressed and more able to manage future episodes of distress, are provided through the person in distress’s contact with the service and its workers. Key methods of change for those in distress included: motivational interviewing; reduce negative emotions; problem solving; planning coping responses; goal setting; action planning; self-monitoring; implementation intentions; and social support [58–68]. We also drew upon established theoretical models and perspectives such as the diathesis-stress model, the integrated motivational-volitional model of suicidal behaviour, compassion, and techniques within established psychotherapies such as cognitive behaviour therapy [6, 42, 69–75]. To limit repetition the practical applications of these methods are elaborated in the following step describing production of the intervention materials.

### Intervention Mapping Step 4: produce intervention

In this step, we used the outputs from the first three steps of the Intervention Mapping protocol to define the intervention and create the staff training programmes and intervention materials.

#### Distress Brief Intervention

The intervention produced was a two-level response with inclusive eligibility criteria: “adults (18 + years) with an emotional pain which led the person to seek help, and which does not require further emergency service involvement”. The initial Level 1 response is provided by trained non-specialist frontline services including Emergency Departments, Police Scotland, Primary Care and Scottish Ambulance Services. At Level 1 trained frontline services provide a sensitive, compassionate response to the person, ease the person’s distress, assess eligibility and offer referral to a local community-based DBI Level 2 service. The Level 2 response is provided by commissioned and trained third sector services, based in the community, who contact the person within 24-hours of referral and provide person-centred compassionate support, with problem-solving support and personalised distress management planning for a period of up to 14 consecutive days.

The intervention materials and tools, described in Table 8, were produced to support staff to provide different elements of the DBI and enable those in distress to feel less distressed and more able to manage their distress in future. The training programmes produced to enable frontline and third sector services to execute the DBI Level 1 and Level 2 responses are described in Tables 9 and 10.

**Table 8:** Description of Distress Brief Intervention materials and tools

**∙** ***DBI Level 1 Referral Form and Service Information Leaflet*:** For Level 1 staff standard forms were produced to facilitate the efficient transfer of referral information across different agencies to the DBI Level 2 service. **∙** ***Standard Operating Procedures for Responding to a Referral*:** Critical to the referral response was initiating contact with the person within 24 hours. Written Standard Operating Procedures were produced to ensure consistency and reliability of follow-up response initiated by the DBI Level 2 service. These included standard contact and re-contact schedule, with ‘what if’ scenarios.
**∙ *Distress Management Plan*:** The Level 2 response is person-centred and with few mandatory components. Notwithstanding this flexibility a key component is the development of a Distress Management Plan (D-MaP), a personal planning resource containing problem solving, coping and solution-focused activities with short and longer-term scope to help the person manage their immediate and future distress. The D-MaP is designed to be used flexibly, allowing for person-centred support and a wide range of experiences and presentation types. The D-MaP represents the practical application of several key theoretical methods of change for those in distress and comprises three main parts *(i) Current distress*,* concerns and strengths (ii) Problem solving*,* action planning (iii) Strategies to manage and cope with distress.* It is expected that the D-MaP will be developed and updated over the course of the 14 consecutive days of support. Information governance and sharing agreements enable sharing D-MaP with other agencies, where appropriate, and with consent. **∙ *DBI Toolkit*:** A range of established distress management, coping, and behavioural change techniques and strategies were rendered as easy-to-use tools, support sheets, activities, and tasks that can be introduced during the Level 2 response [58–68, 71, 73, 76]. The Toolkit can be used as standalone resources or to facilitate development of the D-MaP and was designed to help individuals overcome or manage their immediate distress, acquire insight into their distress and contributing factors, and begin to develop plans, skills and techniques for longer-term management of their distress.

**Table 9:** Description of Distress Brief Intervention Level 1 staff training

***DBI Level 1 training***
∙ A training module was produced to enable different frontline services to provide the DBI Level 1 response. The module addressed three intended learning outcomes, standardised across organisations, professional roles and pre-existing competency levels to ensure consistency in the DBI Level 1 response.
***Intended learning Outcomes***
1. Understand distress and contributory factors. 2. Provide a brief and compassionate frontline response to distress. 3. Have knowledge of DBI ‘Level 2’ support, its benefits and how to make a referral.
***Summary of training themes and activities***
∙ Training addressed frontline staff members' understanding of distress and contributory factors as well as supporting acquisition and/or consolidation of skills in providing a brief compassionate and caring response to people in distress within frontline settings. ∙ Practical aspects addressed referring an eligible person to receive the DBI Level 2 response in the community, as well as information about the nature and potential benefits of the support available through the Level 2 response, consistent with the gatekeeper role of the Level 1 response. ∙ Reflective activities and quotes from those with lived experience of distress were incorporated to contextualise the standard training content within organisational roles and individual experience. ∙ Aide-memoires, support sheets and annotated/model referral forms were included. ∙ Learning was assessed via a 10-item multiple-choice question set.

**Table 10:** Description of Distress Brief Intervention Level 2 staff training

***DBI Level 2 training***
∙ A 2-day trainer-facilitated course produced to enable third sector community support workers to provide the DBI Level 2 response. The training course addressed four core intended learning outcomes.
***Intended Learning Outcomes***
1. Understand the rationale and purpose of the distress brief intervention programme. 2. Gain knowledge of distress and its contributory factors. 3. Make use of interpersonal and communication skills to provide a compassionate and empathic response to distress. 4. Learn to effectively apply a range of skills and techniques to manage distress.
***Summary of training themes and activities***
∙ The training provides a more in-depth understanding of the determinants of distress, appropriate responses to underlying trauma and early life adversity, understanding factors that lead to suicide risk/self-harm, as well as supporting the acquisition of new/consolidation of existing skills in providing a compassionate response to those in distress. ∙ Principles and techniques of motivational enhancement, behaviour change and distress management are addressed: including motivational interviewing, cognitive behavioural approaches, planning and coping strategies. These are linked to the DBI Toolkit resources and their practical application to the development of the D-MaP is demonstrated. ∙ Key practical concerns are addressed, including responding to referrals within expected timeframes and ensuring that a person in distress is prepared for ending the 14-day period of support. ∙ Blended training delivery includes completion of a 60 min e-learning module introducing motivational interviewing, followed by a 2-day facilitator-led course including PowerPoint, group discussion, experiential and role-play formats with feedback and reflection. An activity journal, aide-memoires, handouts, and support sheets are included.

#### Pretesting intervention and materials

The DBI and staff training programmes were developed with key decisions presented and discussed at monthly Distress Brief Intervention National Programme Board meetings. Feedback on written drafts of training was provided by Scottish Government policy leads and clinical advisors, clinical psychologists, psychiatrists, other mental health professionals as well as NHS Education Scotland.

Three members of the intervention development team facilitated two test training sessions of the Level 1 and Level 2 training programmes. The purpose of these test training sessions was to (i) train a limited number of staff within a single site, enabling a small-scale implementation of the intervention prior to further increasing of capacity and scale across organisations and pilot sites, and (ii) assess comprehension, acceptance and appropriateness of the intervention and training messages and activities for the intended staff groups. The test session for the DBI Level 1 training lasted 60 min and was attended by *n* = 9 staff from a single hospital, including emergency department, psychiatric liaison nursing and out of hours care staff. The test training session was immediately followed by a 45-minute discussion and feedback session, during which the participating staff discussed the training material, offered feedback, and made suggestions for improvement. The intervention development team made notes throughout the test training and the discussion and feedback session was audio-recorded. The test version of the DBI Level 2 training lasted 2 days and was attended by *n* = 7 third sector community mental health workers who would provide the DBI Level 2 service for one of the four pilot sites. The test training session was audio-recorded, with in-situ discussion, feedback, and suggestions for improvement of the training and intervention materials taking place. Staff participating in the Level 2 test training also recorded global ratings of self-assessed confidence providing the Level 2 response before and after the test training.

### Intervention Mapping Step 5: plan adoption, implementation and sustainability for real-world contexts

#### Training capacity and creation of alternative training formats

The training pretesting described in Step 4 enabled the establishment and delivery of the intervention pathway and intervention. Over the following 4–6 months the intervention development and training team facilitated further DBI Level 1 and Level 2 training sessions for staff. Wider adoption and implementation within and across organisations required an increase in training activity and rendering of training to different formats that would fit with organisational training plans and systems. In total we produced three different delivery formats of DBI Level 1 training, each lasting approximately 60 min, and which are described in Table 11.

**Table 11:** Different formats of Distress Brief Intervention Level 1 training

**∙ *E-learning module*:** Produced with an e-learning content developer, the development of the e-learning module was iterative, starting with structural and style elements before incorporating the learning material and creating opportunities for learners to engage and use the content effectively. All complementary training resources (handouts, aide-memoirs, examples of information sheets and referral forms) were incorporated and available for direct download and printing. The e-learning module underwent multiple rounds of review and feedback with Level 1 partners, members of the Distress Brief Intervention National Programme Board, Scottish Government, health literacy and accessibility experts, as well as national stigma organisations and those with lived experience of distress. Learning content was refined and navigation, accessibility and functionality was improved. Access to the e-module was via webpage which provided information about the training and learner eligibility, before routing learners to the e-learning platform where an account was created and the DBI e-learning module was allocated.
**∙ *Facilitated group module*:** Produced for local or organisational leads to deliver to groups of staff and comprising a written booklet addressing the standard core learning material, PowerPoint slides for facilitators to introduce the intervention, training and facilitate discussion and reflection around activities. Facilitator guidance was included to enable local or organisational leads to deliver training within the local area or organisation.
**∙ *Facilitated module for Police Scotland*:** Produced for Police Scotland training leads to deliver all standard core learning material using PowerPoint and facilitated discussion. Facilitator guidance was included to enable Police Scotland training leads to deliver training.

Following the initial testing of the DBI Level 2 training, and four further 2-day training sessions, a ‘DBI Level 2 Practitioner Training: Delivery Pack’ was created to support a train the trainer model of delivery that would enable third sector organisations providing the Level 2 response to deliver training locally and ensure timely access to training based on turnover and growth in the teams. The DBI Level 2 Practitioner Training: Delivery Pack included all training material and resources, alongside comprehensive guidance on the sequence, structure, timing and facilitation of the training. Testing this resource took place over two days, with two local leads using the resource to train nine new members of staff, observed by a member of the intervention development and training team. Observer notes and feedback from the leads who delivered the test session were used to refine and finalise the training.

#### Staged implementation and upscaling

A staged approach to implementation and upscaling was adopted. Within each of four pilot areas a limited number of staff providing the DBI Level 1 response were trained using the preferred training format, with a 2-day DBI Level 2 training session held for the local third sector community support organisation providing the DBI Level 2 service. This enabled the setup of the intervention and pathway in each pilot area, while at the same time limiting demand within each pilot area during the early stages. This was followed by a period of incremental upscaling, supported by local or organisational delivery of training to staff to increase capacity within each of the four pilot areas. Following this model of staged implementation and upscaling, at the end of the development phase there should be sufficient capacity for an independent evaluation of the implementation of the pilot intervention programme. Monthly reports detailing use of the DBI Level 1 e-learning module were compiled and distributed to local or organisational leads to monitor local implementation of training.

### Intervention Mapping Step 6: evaluation of development phase implementation

An independent evaluation of the DBI pilot programme, adopting a realist approach, was commissioned by the Scottish Government with the aims of understanding whether the programme was implemented as intended and determining its impact on services, practitioners and individuals [77, 78]. The independent evaluation commenced following the completion of the development phase defined by the current work, with the findings reported elsewhere [77, 78]. The evaluation plan for the development phase of work reported on here is therefore focused on the implementation of the DBI Level 1 and Level 2 training and reports on the number of staff trained and comparisons of mean self-assessed confidence ratings obtained immediately before and after training completion using paired sample t-tests.

During the development and controlled implementation phase a total of 525 staff were trained across the four pilot areas. This included 472 staff in frontline service roles (*n* = 218 police officers, *n* = 254 health care roles including emergency department, primary care and ambulance services) who were trained to provide the Level 1 response, and 53 third sector community-based support workers trained to provide the Level 2 response. Of those trained to provide the Level 1 response, 35% were trained using the e-learning module and 65% were trained using one of the facilitated versions of training. Immediately before and after training, staff participating in Level 1 training reported self-assessed confidence in their knowledge and skills, on a scale of 1 (low confidence)– 10 (high confidence) across four statements linked to the intended learning outcomes of the training. Staff participating in the 2-day Level 2 training session also reported self-assessed confidence (1-10) in relation to a single global measure. Self-assessed confidence ratings obtained immediately before and after training were available for 71% (*n* = 335) of Level 1 frontline staff trained during the development and implementation phase and all Level 2 staff trained. Paired sample t-tests carried out on pre- and post-training mean ratings indicated that there were statistically significant increases in confidence observed across each indicator (Table 12).

**Table 12:** Comparison of mean (SD) confidence ratings provided by Distress Brief Intervention Level 1 and Level 2 staff immediately before and after training completion

	Pre-training Mean (SD)	Post-training Mean (SD)	t
**Distress Brief Intervention Level 1 staff (** ***N*** **= 335):** How confident do you feel in your knowledge/skills:			
*…understanding distress and contributory factors?*	6.10 (2.21)	8.46 (1.36)	21.38***
*…providing a brief compassionate frontline response to someone in distress?*	6.59 (2.21)	8.53 (1.34)	17.83***
*…referring an eligible person in distress to the DBI Level 2 service?*	3.70 (2.80)	8.36 (1.68)	29.91***
*…awareness of DBI Level 2 support and its benefits for a person in distress?*	3.45 (2.68)	8.46 (1.51)	33.0***
**Distress Brief Intervention Level 2 staff (** ***N*** **= 53):** How confident do you feel:			
*…about delivering Distress Brief Intervention?*	6.11 (1.93)	8.65 (1.13)	9.86***

t: paired samples *t *Test

***p < 0.001

## Discussion and current status of the Distress Brief Intervention

**Fig. 2:**
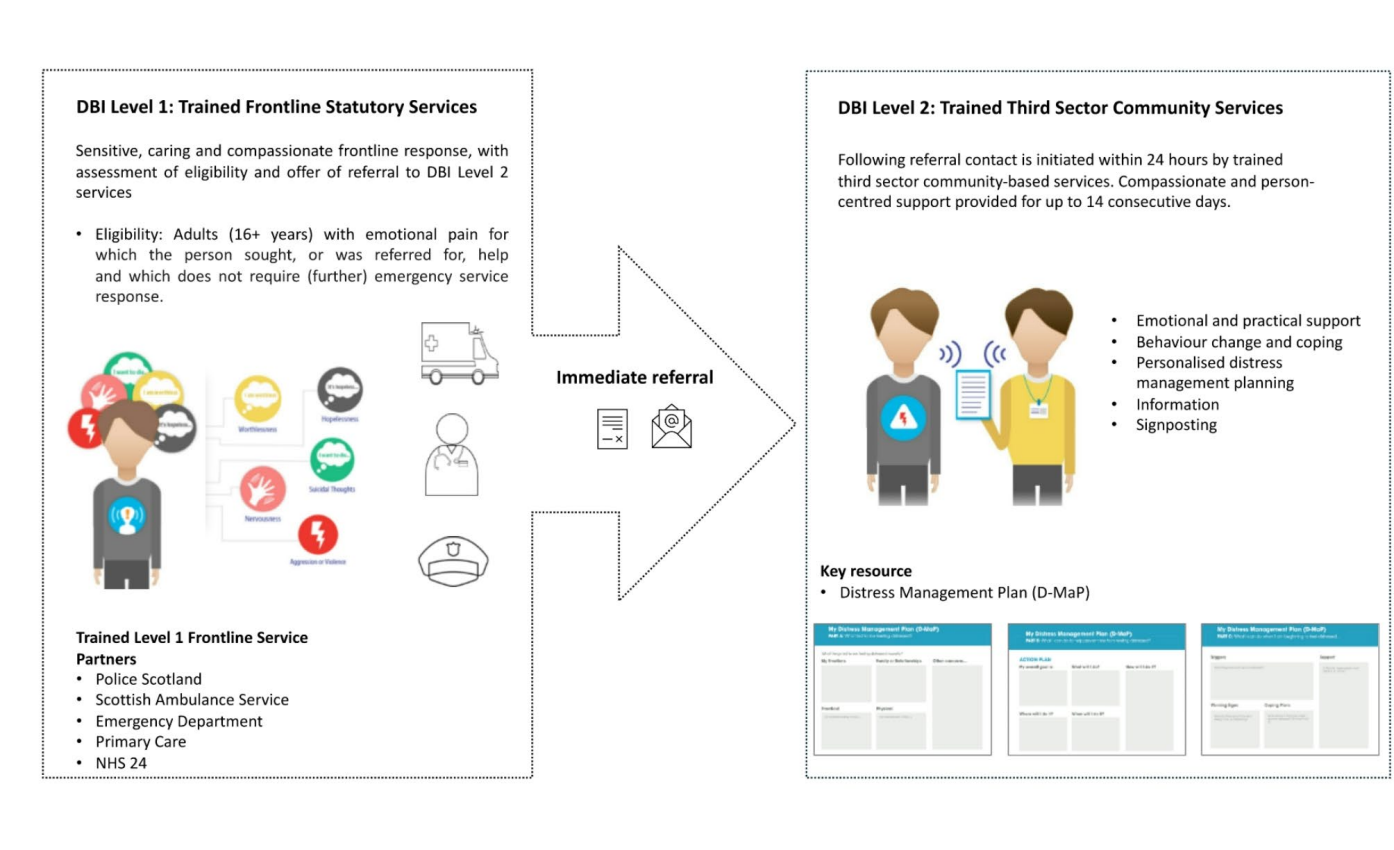
Distress Brief Intervention (DBI): a multi-agency service to provide connected, compassionate support for people in distress

We have reported the systematic development and implementation of a national Distress Brief Intervention (DBI), a first intervention of its kind internationally. Brief interventions for distress are scarce which may reflect the challenging nature of carrying out rigorous intervention development activities for complex behaviours and populations. The DBI is a time-limited, two level, complex intervention for adults experiencing emotional distress. It offers a coordinated response across a range of frontline statutory services including primary, acute and ambulance healthcare, as well as police and third-sector community organisations in Scotland. Intervention components include competency-based training programmes for staff, a simple and effective referral pathway, standard operating procedures, guidance for intervention providers and a range of distress management, behaviour change, coping and planning toolkit resources for use with those in distress. Training programmes were developed to address the knowledge, skills and attitudes necessary to provide a brief compassionate response to distress and enable consistency of response across different organisations and presentations of distress. The training programmes were tested and refined as part of a staged implementation in four pilot areas, with a total of 525 staff trained to provide the intervention during development and implementation phase. Figure 2 presents an overview of the DBI, inclusive of a lower eligible age range (16 + years) and an additional frontline service partner (NHS 24) introduced following the initial development of DBI.

Although the DBI is novel several intervention components and principles are shared by other well-established distress responses. For example, offering person-centred practical care and support, listening and information, including connections to further support services through non-specialist staff at a point of presentation overlaps with Psychological First Aid [21, 22]. However, there are also clear differences, including the applicability of DBI to those in distress irrespective of precipitating circumstances or events. In contrast, Psychological First Aid has tended to be used immediately following the occurrence of a critical incident, emergency or disaster as a means of mitigating an acute stress response to extremely threatening traumatic events. The DBI, by design, connects the immediate frontline (Level 1) response with further community-based support (Level 2) lasting up to a further 14 consecutive days. Thus, DBI is generally applicable to a wide range of distress presentations which may or may not have been precipitated by a critical incident, as well as offering a more extended period of support lasting up to 14 consecutive days away from acute settings.

The Intervention Mapping protocol enabled a structured and explicit approach to development activities, but the limited time available to develop the intervention and carry out the initial implementation led us to take an adaptive approach to protocol adherence. For example, for two of the three programme outcomes, the most efficient approach was to develop training competency frameworks for the Level 1 and Level 2 training programmes and specify performance objectives as the competencies expected of staff following training; with knowledge, attitude and skills as the key determinants of these competencies. In turn we identified the changes in these determinants needed to meet the competencies. Thus, we adapted the second step of the protocol to more closely align with the focus of these two programme outcomes and our experience of applying the Intervention Mapping protocol to complex multi-dimensional behaviours is similar to that of other researchers who have cited the substantial time and resources needed to adhere to the protocol [48, 79, 80].

A strength of the intervention is its strong basis in formative work and engagement directly with staff and organisations who will deliver the intervention and those with a lived experience of distress. Without the strong support and engagement of the statutory and third sector partner organisations it is unlikely that the novel DBI would have been developed in this timeframe. For example, the partner organisations facilitated our access to staff and those with lived experience of distress, provided suggestions and feedback on training and associated resources, participated in multisectoral development work and contributed to the governance and decision-making structure through the Distress Brief Intervention National Programme Board. The strong national approach to the endeavour alongside significant resourcing for the development of the DBI and its multi-site implementation, including support for the coordinating and strategic role provided by a Distress Brief Intervention Central Team, comprising programme manager, analyst and administrative support cannot be overstated.

## Current status of the Distress Brief Intervention

The development of the intervention and initial implementation in four pilot areas took place over an 18-month period between September 2016 and March 2018. Since then, the DBI has evolved, been independently evaluated and expanded into a national service, as described in Table 13.

**Table 13:** Development and expansion of the Distress Brief Intervention

∙ **Spring/summer 2019:** the eligible age range for DBI was lowered from 18 to 16 years, supported by additional training adopting a young person perspective for staff providing the DBI Level 1 and Level 2 response.
∙ **2019:** following development and piloting in four areas of Scotland, expansion to other sites in Scotland began through the DBI Associate programme, providing full access to intervention, training and infrastructure resources to administrative regions of Scotland.
∙ **April-2020:** in response to potentially high levels of distress associated with the Covid-19 pandemic, and the inaccessibility of existing in-person support and services, a new pathway providing national telehealth coverage was created. The pathway enabled eligible people in distress contacting NHS 24, to receive a referral to the DBI Level 2 services. To facilitate the additional referral pathway and shift to providing telehealth support during the pandemic we developed further training for the new DBI Level 1 partners and additional facilitated training sessions and distress management toolkit resources for DBI Level 2 providers.
∙ **January-2021:** we conducted scoping work which led to recommendations provided by a national Children and Young People Advisory Group, to establish a small-scale ongoing pilot of DBI for those aged 14 years and over. This pilot introduced a novel tripartite model, with school pupil support/pastoral teams having the option to refer pupils in distress aged 14 years and over to receive support from a DBI Level 2 Children and Young People’s service. The third part of the model links schools and the DBI with local CAMHS teams, enabling escalation or de-escalation to ensure the appropriate level of care is provided.
∙ **2022:** an independent evaluation of the DBI pilot programme, adopting a realist approach, reported on programme implementation, its impact on services, practitioners and individuals, and provided recommendations [77]. A second independent evaluation reported on the extended programme over the Covid-19 pandemic [81].
∙ **2023–2025:** two further evaluations of the DBI programme of work are ongoing—an independent evaluation of DBI for children and young people, and an evaluation to understand the possible suicide-protective effects of DBI [82].
∙ **2024:** the DBI is rolled out nationally, with all 31 health and social care partnerships in Scotland providing DBI or an aligned service.

Following the expansion of the DBI, as of January 2025, more than 80,000 referrals for adults in distress have been made using the DBI. The DBI is supported by more than 3,800 members of health and social care staff in statutory frontline and third sector community support services who have been trained to provide the appropriate DBI response. The DBI has been rolled out nationally across the whole of the country such that each of 31 health and social care partnerships in Scotland provide a DBI or aligned service. DBI is also being piloted in England [83]. In addition, the DBI (re-named Distress Brief Support) is also being trialled in Australia and has been included in Victoria’s Royal Commission on mental health services [84] as well as Australia’s ten year (2025–2035) national suicide prevention strategy [85].

## Limitations

During the formative work undertaken with stakeholders in the development of DBI, we were unable to engage staff representing all frontline delivery partners to participate in interviews or focus group discussions. This led to a greater reliance on consultations and guidance provided by service leads whose experiences and views on the DBI may not be representative of those staff routinely responding to distress. We also did not collect detailed demographic information from those who contributed to the interviews and focus groups. The intervention development phase reported here also took place over a relatively short period– 18 months from 2016 to 2018– and our analysis of the interviews and focus groups prioritised themes directly relevant to our development aims rather than being more exploratory. The development also predated the COVID-19 pandemic, which has significantly altered the availability and accessibility of mental health and other vital health and social care services [86]. Even still, the DBI in Scotland has successfully adapted to changes in services and service use, including a new pathway via national telehealth services and hybrid support from community services. Evaluation of the staff training programmes relied on self-assessed confidence ratings reported by those taking part in training and alternative assessment methods could offer more precise and robust assessment of key competencies.

## Conclusion

A multi-agency national Distress Brief Intervention was systematically developed and implemented in a range of non-specialist frontline and community settings in Scotland. Up-take of training and evaluations of training indicate it is highly acceptable to potential providers and improves key competencies. Following independent evaluation, the Distress Brief Intervention has been rolled out nationally across the whole of Scotland, and has significant potential as a model of care and prevention internationally, including countries with low statutory health resources.

## Supplementary Information

Supplementary Material 1. Additional File 1 Interviews and focus groups with (i) those with lived experience of distress and frontline services use (ii) staff experienced in responding to distress. Additional File 2 Figure S1. Distress Brief Intervention Programme: Theory of Change [50] with focal outcomes for the development phase highlighted. Additional File 3 Table S3. Performance objectives/ competency statements, determinants and change objectives for programme outcome: Distress Brief Intervention Level 1 frontline staff have the skills, competencies and confidence to deliver a Level 1 intervention; Table S4 Performance objectives/ competency statements, determinants and change objectives for programme outcome: Distress Brief Intervention Level 2 practitioners have the skills and competencies to deliver a Level 2 intervention.

## Data Availability

The datasets used and/or analysed during the current study are available from the corresponding author upon reasonable request.

## Abbreviations

A&E: Accident and Emergency /Emergency Department
CAMHS: Child and Adolescent Mental Health Services
DBI: Distress Brief Intervention
GP: General Practitioner
NHS: National Health Service
