# Supplementary material for: Development of a national Distress Brief Intervention: a multi-agency service to provide connected, compassionate support for people in distress
Source: BMC Health Serv Res. 2025 Apr 4;25:478. doi: 10.1186/s12913-025-12469-3 (PMC11970026; doi:10.1186/s12913-025-12469-3)
Supplement: Supplementary file 1 — Supplementary Material 1. Additional File 1 Interviews and focus groups with (i) those with lived experience of distress and frontline services use (ii) staff experienced in responding to distress. Additional File 2 Figure S1. Distress Brief Intervention Programme: Theory of Change [50] with focal outcomes for the development phase highlighted. Additional File 3 Table S3. Performance objectives/ competency statements, determinants and change objectives for programme outcome: Distress Brief Intervention Level 1 frontline staff have the skills, competencies and confidence to deliver a Level 1 intervention; Table S4 Performance objectives/ competency statements, determinants and change objectives for programme outcome: Distress Brief Intervention Level 2 practitioners have the skills and competencies to deliver a Level 2 intervention. [file 12913_2025_12469_MOESM1_ESM.docx]

**Additional File 1. Interviews and focus groups with (i) those with lived experience of distress and frontline services use (ii) staff experienced in responding to distress.**

Interview/focus group topics and questions for those with lived experience of distress

1. *Factors influencing seeking help*

- What led up to the point when you sought (*or received*) help for how you were feeling?
- Had this been going on for a while or was it quite sudden?
- Had you tried to speak to anyone else before this?
- Who did you approach and why did you seek help?
- How were you feeling when you approached the frontline service?

1. *Experiences during frontline service use*

- How would you describe what happened when you sought help?
- How aware were you of what was happening and the care/support you received?
- How did the person or team you were speaking to seem to be towards you and what you were experiencing?
- Did you feel you received the help you needed from the frontline service?

1. *The role of frontline services in helping people in distress to access support following the acute episode of distress*

- What happened later after you had sought help?
- Could the frontline service have done anything differently?
- What further support did you get afterwards, if any?

1. *Support that could be offered after attending a frontline service because of distress*

- Were there things you didn’t feel you could talk about to the person or service you attended when you were in distress? If yes, what made you feel this way?
- How would you have felt if the frontline service had been able to make a referral for you to see/speak with someone for some further support immediately after you had gone for help?
- What kind of support do you think might have been helpful?

1. *Views on the proposed Distress Brief Intervention [Provide the person/group with a short explanation of the proposed intervention]*

- Do you think there are good reasons for having a service like this available to people who are experiencing distress? How might it help?
- How would you feel about being offered this kind of help?
- Would there be any barriers to getting further support from 3^rd^ sector services? How might such barriers be overcome?
- What would be important for a service like this to include?
- How should it be provided (face to face, telephone etc)?
- How long should the support be offered for and how often do you think it would be helpful to get the contact from the service?

Table S1. Additional quotes from interviews and focus groups conducted with those with lived experience of distress and frontline services

| *Experiences of service use and seeking help by those in distress:*   - *“You get medical staff who are just there to fill in your chart, and you can get other ones that come over to you and are like, how are you feeling now, sort of attitude.”* [Person with experience of distress and contact with frontline services; interview] - *“They were talking to me in the ambulance. Obviously, one of them was driving, the other was in the back. And they were talking to me, and saying to me, you know, what made you want to do this, you know, asking me about my family, asking about my situation, writing down information. So it made me feel like they cared, and they actually, you know, wanted to help me.”* [Person with experience of distress and contact with frontline services; focus group] - *“So once my partner came back, they just left me. I was just left, oh there's your partner, you won't do anything because your partner's there. And they just left me. And I had to go and seek help again, myself, after that. So that wasn't the best...”* [Person with experience of distress and contact with frontline services; focus group] |
| --- |
| *Views on a Distress Brief Intervention:*   - *“ I think it’s a great idea, I think you’ve got the best of intentions, I just don’t think you realise what you’re up against, if that makes sense?”* [Person with experience of distress and contact with frontline services; interview] - *“Because they’ve got the support there within the 24 hours rather than waiting a week. A lot of people find themselves in crisis and they don’t know where to turn. So if they know that they were going to be referred by frontline services to a service that’s going to actually help them, that could hold off taking their own life.”* [Person with experience of distress and contact with frontline services; focus group] - *“Different levels of distress have you in different emotional states so like I was in a high level of distress so I couldn’t say that that would have been beneficial at the time because it’s like...you’re trying to put out a fire with something that’s not going to do it. It’s a fine line to...it’s a fine judgement call I think. It depends on the situation. You can’t just say that it would help everyone in that situation…….. If somebody says they’re going to like kill themselves the now, just for an extreme example, then you can’t say, oh, I’ll phone you back tomorrow.”* [Person with experience of distress and contact with frontline services; interview] - *“Being listened to. Because if you have somebody to talk to, that can be all that is needed to stop a crisis from going any further.”* [Person with experience of distress and contact with frontline services; focus group] - *“People just don't open up right away, it takes some time, it's like everything else you don't tell everybody right away all about yourself, you know what I mean, the first day. If you're wary of the particular person you've got defences and it takes a wee bit time before you start to kinda open up a bit and tell them so much exactly how you're feeling. So you can't just take, you know, talking 14 days, it could be fine, that could work but sometimes, as you say, it could last longer because it takes time for people just to kinda open up in general.”* [Person with experience of distress and contact with frontline services; focus group] - *“Because there will be a monumental demand on this service if it's put in place. And I just wonder if you're prepared for that as well, because there isn't anybody else doing something like this.* [Person with experience of distress and contact with frontline services; focus group] - *“But then the drop-off point after the two weeks of help, if you're still needing help, then you've got cases that…who plugs the gap for that. Then the gap's still here whether you get referred or not and you're still in distress potentially, I don't know.”* [Person with experience of distress and contact with frontline services; focus group] - *“To be able to just tell one person that, it’s like when you go to a doctor and you get a different doctor and they’re like, right, what’s wrong with…do you know my history, you’re actually saying, do I have to tell you all this again or do you have to read notes. And I’d rather someone kind of understood me from beginning to that end 14 days than to have to tell someone else or wait for someone else to read the notes on me to understand that. And again it’s building that rapport with someone that they get a sense by as you’re going along that journey just exactly how you’re feeling and who you are and what you might need, and it might be ten days in you think, you know what, I actually think this might help because I know you a little bit better now that we’ve tried this and how would you feel about this”.* [Person with experience of distress and contact with frontline services; focus group] - *“It doesn’t matter how many hours of training you do, that’s not their job, and a lot of them really despise it. And, it doesn’t matter if it’s your first mental health crisis, and these police officers come out, and if they’re really bad to you, because at the end of the day, they didn’t join the police to be a mental health nurse, they joined the police to lock people up…Like, as much as you try and train them, as much as you try and change the system, frontline staff are always going to be annoyed.”* [Person with experience of distress and contact with frontline services; interview] - *“The NHS Community Service is like, the CMHT, they will dump people on you continuously, continuously, and people will get referred continuously...”* [Person with experience of distress and contact with frontline services; interview] - *“I think if you’re going to train people, getting people in that have had experience, like people that, make it more human, anyone can sit down and read, like it doesn’t connect you with anything, it doesn’t make you feel anything”* [Person with experience of distress and contact with frontline services; interview] |

Interview/focus group questions and prompts for staff experienced in responding to distress

1. *Current practice*

- How do you currently approach people who seem to be experiencing a high level of

distress? Are there set procedures involved? Is there any time pressure? Are there environmental factors you need to consider?

- In your experience what works well? Can you offer examples?

1. *Views on the proposed Distress Brief Intervention [Provide the person/group with a short explanation of the proposed intervention]*

- Do you view what we’re aiming to do with this approach as being part of your role? If yes, why? /If not, whose role is it?
- How confident do you feel about using this in practice?

1. *Potential benefits of the proposed Distress Brief Intervention*

- What do you see as the potential benefits of this type of intervention to those in distress? Immediately/during the episode? In the future?
- And what about the benefits to you in your role as someone who responds to those in distress? Immediately/during the episode? In the future?

1. *Potential challenges to implementing the proposed Distress Brief Intervention*

- What do you see as the key challenges to a successful implementation? As someone who provides a frontline response to distress? When considering this more widely as part of the process and systems which [agency/service] operate within?
- What do you see as the solutions, what will help to ensure a smooth implementation?
- Are there aspects they feel uncomfortable or uncertain about?

1. *Inter-agency working (optional topic, time permitting)*

- How can [agency/service] and other agencies or community services work together to deliver the proposed Distress Brief Intervention? Communication links? What resources are available?
- Can you take action directly or are further consents needed?

Table S2. Additional quotes from interviews and focus groups conducted with staff experienced in responding to distress

| *Potential benefits of Distress Brief Intervention:*   - *“It could be really preventative in the fact that you go to hospital, you wait the six hours and sometimes you've reached rock bottom and you want somebody to say 'I hear what you're saying, that must be really, really hard for you, here's what I can do for you' which is very limited but if you can say 'a worker's going to contact you in 24 hours to see how and if they can help you' that's something for that person to hang onto, so in terms of risk management, it's helping them because they'll hang on because they know that's coming, but it's also preventing them re-presenting with the same issue when those issues haven't been resolved, and in that 14 days so much will be able to be achieved rather than in that maybe 45 minutes, if it's even that, the amount of time that you spend with the frontline workers in the hospital room, so I think it'll be really valuable.”* [Third sector mental health support worker; focus group]” - *“you're chaotic and you're everywhere, so that person's holding the space for you and offering you some wee bullet points of structure that you can then hang onto and it's a framework for your journey to get back to whatever normal is for each person.”* [Third sector mental health support worker; focus group] - *“You know, there's nothing negative about this. I think you get a conversation going, you're always going to get, well, I'm going to tell you this and blah blah blah, and that's natural, and that's the reason you're here. But every little wee bit that can help us, that we can say to somebody, look… before we were just walking out of somebody's house, while not having brought it to a total conclusion, we can now say, right, here's a card, you're going to be getting a phone call in 24 hours..”* [Police Scotland officer; focus group] |
| --- |
| *The interplay between the Distress Brief Intervention and one’s current role*  *“from what we do already from the initial visits, from the assessment four to five week assessments we do with people, obviously this is mentioned here your 14 days and you've said yourself that could vary, it's really listening to people what, in a person centred manner, what they actually require, what they need, what we can then signpost to people from what our experience we've got of signposting.”* [Third sector mental health support worker; focus group]   - *“Our role never changed, it’s there to safeguard and introduce people into the right agencies, so that they’re getting the help that they actually need. I think once we’ve instigated that and that first role of introducing them, for me in an ideal world, there should be personnel at the hospital that’s meeting these people, and then you’ve got a secure set-up that’s willing and able to safeguard them whilst they’re there. If they leave, then it’s their responsibility to be sure of where they’re going.”* [Police Scotland officer; focus group] - *“…if it's suicidal thoughts…, they don't want to be here, it's probably still, well, certainly would still be taking them to a health professional to make that assessment.”* [Police Scotland officer; focus group] |
| *Potential challenges/barriers to the implementation of the Distress Brief Intervention:*   - *“I wonder about attitudes having to change in terms of we are third sector workers and we come across, I personally I'll not say we, have come across situations where you're not very respected in the same manner I would be if I said I'm a policewoman or I'm a doctor; so on those occasions you've been your service user's voice because at that point they're at such a crisis they require you to be their voice, they'll take up their voice again when they can but I've come across situations where it's... and I've been upset about it 'well what d'you know?' and I explain 'I'm speaking to you as a lay person, I do have training to do this job but right now I'm speak to you as a person who knows this person and I know that I'm very concerned and I'm handing it over to you because I feel it's gone beyond my capacity as a worker and it's now your role”* [Third sector mental health support worker; focus group] - *“if we are involved too long we extend that person's period of unrest and distress because we're reminding them sometimes, so there comes a point where we say to them 'd'you know what, we've got this plan, how d'you feel about ending it, it doesn't mean that I'm gone or we're gone, we're still here, but you can phone us if you need to' and a lot of the time they'll say 'well yes, actually I'm ready to end the plan' and it's their decision. But I think some people you could cause you're offering them support, you could run it on forever and they would allow you to do that but then that's not good for them.”* [Third sector mental health support worker; focus group] - *“I wonder about attitudes having to change…I personally, I'll not say we, have come across situations where you're not very respected in the same manner I would be if I said I'm a policewoman or I'm a doctor”* [Third sector mental health support worker; focus group] - *“Obviously it's going to be an initial small service referring people on, if it goes to a massive scale where there's that much people coming through the referral system, I mean, obviously it's going to have to be a bigger support on the third sector to be able to support all that, which is a positive thing for any support agency because obviously if it's supply and demand, you've got to have the service there to support what's coming through and be able to adapt…”* [Third sector mental health support worker; focus group] - *“So they've tried to phone her, can't get in contact with her. I'd imagine they've tried to phone her once, twice maybe. And the default is phone the police.”* [Police Scotland officer; focus group] - *“no-one can share anything with anyone, really, and so you’ve got the police that are running around, you’ve got third sector, like voluntary organisations trying to support, not knowing what’s going on, you’ve got psychiatry, and no-one’s speaking to each other, and you’ve got a person that can go from pillar to post and back and forth and round and round, and everyone’s got high level involvement, and it’s all this resources, and if everyone could just speak to each other with the person and have a bit of consistency, so I don’t know if DBI would come into being able to help with that, or rather it’s not going to be at that level…”*[Third sector mental health support worker; focus group] - *“… like being used almost as like a dumping ground, sometimes…now and again, like a service will almost pass somebody over when they’re a service we would refer them back to or signpost them to, and sometimes, I hate to be cynical, but sometimes it’s almost as if they’re like, ugh, go and speak to these people, there’s the number, off you go, it’ll be fine, go on…”* [Third sector mental health support worker; focus group] - *“On paper it's fine, the idea's great, ideal, aye it's perfect, but it's just having that safety net for cops to trust the system and not be done over in a couple of years’ time when somebody dies and it's been us that went out and dealt with them and we're the ones that'll catch it.”* [Police Scotland officer; focus group] - *“No doubt it'll be a Moodle online course and they're the most mind numbing courses ever and as soon as one person does it then that person will just crowd round everybody else and tell them the answers and we just flick through the training.”* [Police Scotland officer; focus group] |
| *Potential facilitators to the implementation of the Distress Brief Intervention:*   - *“if we’re working with somebody who’s really struggling to attend appointments we’ll say, why are you struggling, what can we do to get you attending appointments, and maybe attend an appointment with them goal is always, they’ll attend appointments unsupported and it’s always clear that we’re not a service that’ll attend appointments with somebody, but we work, we do everything we can to get the person developing the skills and the confidence to go to these appointments.”* [Third sector mental health support worker; focus group] - *“It’s about confidence in what the remit is and what we do and what we don’t do, and about clarity, like making sure that it’s very clear, as opposed to the referring agencies, what it is, I suppose, just making sure that the information, the training, you know, all this is very kind of clear”* [Third sector mental health support worker; focus group] |

**Additional File 2.**

Figure S1. Distress Brief Intervention Programme: Theory of Change (45) with focal outcomes for the development phase highlighted.


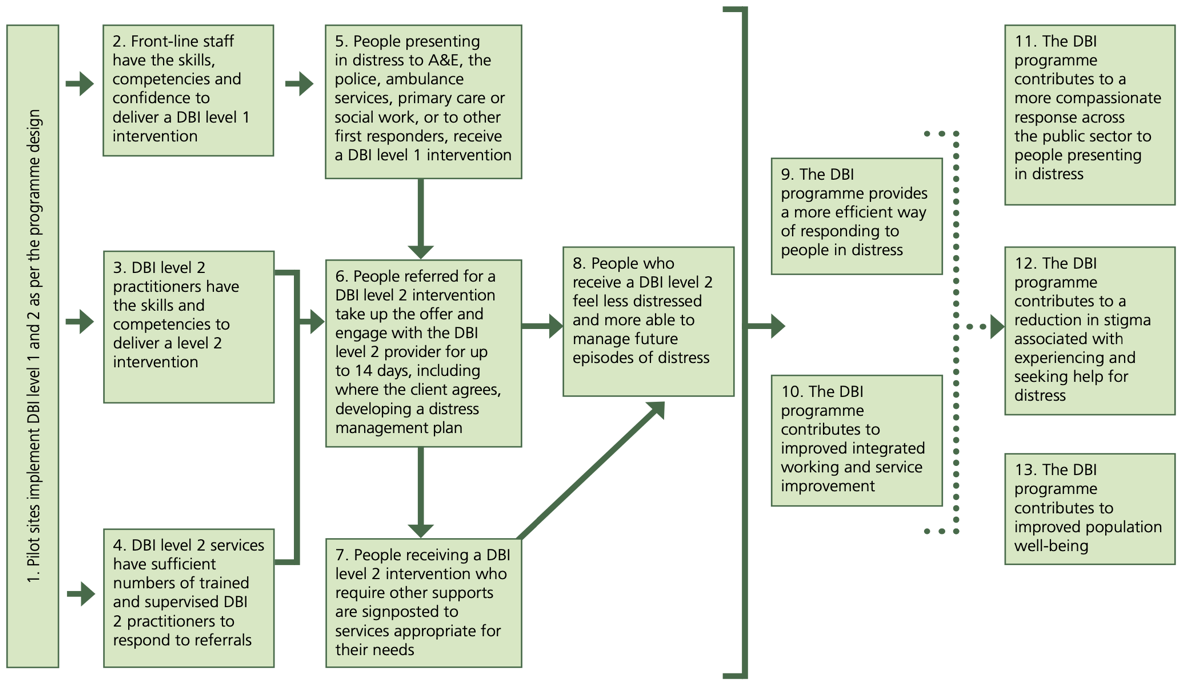


**Additional File 3.**

Table S3. Performance objectives/ competency statements, determinants and change objectives for programme outcome: Distress Brief Intervention Level 1 frontline staff have the skills, competencies and confidence to deliver a Level 1 intervention

| **Distress Brief Intervention Level 1: Knowledge, awareness, context** |  |  |
| --- | --- | --- |
| Performance objectives / competency statements | Determinant | Change objectives |
| **1.1 Understands the rationale and purpose of the distress brief intervention programme** | Knowledge  Knowledge  Knowledge  Knowledge | - 1. **Distress brief intervention**   Understands what is meant by distress and distress brief intervention  Is aware of national policies relevant to distress and brief interventions (Suicide Prevention, Mental Health, Recovery)  Understands the aim and design/method/approach of the DBI programme  Understands the importance of inequalities in relation to mental health and wellbeing and how it applies to understanding of distress |
| **1.2 Reflects on feelings and attitudes to distress and appreciates their potential influence on delivery of distress brief interventions** | Attitudes  Knowledge  Knowledge | - 1. **Attitudes to distress**   Recognise their own and others’ feelings and opinions about distress  Understands that different feelings or attitudes about distress might influence their approach and practice to distress brief interventions  Awareness of important influences on distress (e.g. individual, social, cultural and environmental), including stigma |
| **1.3 Knowledge of and ability to act within professional and ethical guidelines** | Knowledge  Attitudes  Knowledge  Knowledge | - 1. **Professional practice**   Awareness of relevant regulatory frameworks (e.g. child and adult protection)  Commitment to rights-based approaches in mental health  Record and manage sensitive data in line with agreed protocols  Understands importance of ethics in their role and act accordingly |
| - 1. **Understands the importance of maintaining personal wellbeing and exercising relevant self-care actions** | Knowledge  Knowledge | - 1. **Self-care**   Aware of ways in which practitioner role can adversely impact upon personal health wellbeing  Aware of support options available to maintain their own wellbeing |
| **Distress Brief Intervention Level 1: Identification and assessment** |  |  |
| Performance objectives / competency statements | Determinant | Change objectives |
| **2.1 Ability to recognise that distress may present in various forms** | Knowledge & Skills  Skills | **2.1, 2.2, 2.3 Recognising distress**  Recognise that distress may present in various forms, including medically unexplained symptoms  Makes the most of opportunities as they arise by asking relevant questions to probe for possible distress |
| **2.2 Ability to engage in conversations about mental wellbeing and distress clearly and sensitively** | Knowledge  Skills  Skills  Skills & attitudes  Skills & Attitudes  Skills  Skills  Skills  Skills  Skills | **2.2, 2.3 Addressing the issue**  Recognises appropriate opportunities to discuss distress  Creates an environment conducive to discussions of mental wellbeing  Raises issues around distress carefully, monitoring the impact on the person in distress and adapting approach as necessary  Informs importance and relevance of discussing distress  **2.2, 2.3 Interpersonal and communication skills**  Uses language which is appropriate for the person in distress and checks for understanding  Maintains appropriate levels of eye-contact and offers person in distress full attention  Employs *open-ended* questions to probe and elicit information  Uses *affirming* statements to person in distress  Uses *reflective listening* skills during interaction with person in distress  *Summarises* important parts of the interaction |
| - 1. **Ability to undertake an empathic assessment of the individual** | Knowledge & Attitudes  Knowledge & Skills | **2.3 Aware of value and importance of empathic assessment**  Understands the value, and skills involved in an empathic frontline assessment of a person in distress:  *Value and relevance of empathic assessment:*  *establishes rapport, enabling efficient understanding of relevant history, including factors related to current episode of distress; recognition and validation of person in distress’s feelings, motivations and intentions; encourages person in distress to become actively engaged in decisions around their care and support;* *prediction of effects of staff actions on person in distress, including decisions around suitable support/help and avoidance of negative or hostile reactions; enhanced awareness of factors influencing distress contemporaneously, including environmental stressors (e.g., noise, lighting), staff demeanour and approach*  Understands how to undertake empathic assessment in context of a frontline response to distress |
| **Distress Brief Intervention Level 1: Delivering Distress Brief Intervention** |  |  |
| Performance objectives / competency statements | Determinant | Change objectives |
| - 1. **Understand the importance and how to enact a compassionate frontline response for distress** | Knowledge & Skills  Knowledge & Skills | **2.3, 3.1 Compassionate response**  Understands the importance, key skills and attributes of a compassionate frontline response  *Skills: Attention; imagery; reasoning; behaviour; sensory; feeling*  *Attributes: motivation/care for wellbeing; sensitivity; sympathy; distress tolerance; empathy*  Understands how to use compassion skills and attributes in the context of a frontline response to distress |
| - 1. **Provide relevant information and advice about mental wellbeing and distress** | Knowledge & Skills | - 1. **Providing relevant information**   Gives accurate information and guidance clearly and in a way which is appropriate to the individual |
| - 1. **Identify and problem solve when faced with barriers to delivering Distress Brief Intervention** | Skills | - 1. **Barriers and concerns**   Identify and resolve possible challenges to delivering Distress Brief Intervention |
| - 1. **Provide clear and accurate signposting information** | Knowledge  Skills | - 1. **Signposting to other services or supports**   Knowledge of appropriate supports and services  Clear communication and explanation of services and supports in a way which is appropriate to the person in distress |
| - 1. **Make an offer and execute a referral to Distress Brief Intervention Level 2** | Knowledge & Skills  Knowledge | **3.5 Referrals**  Able to explain the purpose, nature and value of referral to Distress Brief Intervention Level 2 for the person in distress  Understand how to make a referral to Distress Brief Intervention Level 2 |

Table S4. Performance objectives/ competency statements, determinants and change objectives for programme outcome: Distress Brief Intervention Level 2 practitioners have the skills and competencies to deliver a Level 2 intervention

| **Distress Brief Intervention Level 2: Contact and assessment** |  |  |
| --- | --- | --- |
| Performance objectives / competency statements | Determinant | Change objectives |
| **4.1 Respond to a referral from a DBI Level 1 delivery agency, ensuring initial contact with the person in distress is made within 24hrs** | Knowledge  Knowledge & Skills  Skills | - 1. **Responding to referrals**   Understand how to respond appropriately to a referral within 24 hours  Introduce the purpose and scope of the Distress Brief Intervention Level 2 support  Explore options for further schedule of contact and support over a 14-day period |
| - 1. **Undertake an empathic assessment, including establishing**   **an individual’s suitability for DBI Level 2 support and whether they are an imminent risk to themselves or others** | Knowledge  Skills  Knowledge & attitudes  Knowledge & Skills  Knowledge & Skills  Knowledge & Skills | **4.2 Recognising distress**  Recognise that distress may present in various forms, including medically unexplained symptoms  Making the most of opportunities as they arise by asking relevant questions to probe for possible distress   - 1. **Aware of value and importance of empathic assessment**   Understands the value, and skills involved in an empathic Level 2 assessment of a person referred for a Level 2 response:  *Value and relevance of empathic assessment: establishes rapport, enabling efficient understanding of relevant history, including factors related to episode of distress which resulted in referral; recognition and validation of person in distress’s feelings, motivations and intentions; encourages person referred to become actively engaged and collaborate with staff during problem-solving contacts ;* *prediction of effects of staff actions on person in distress, including decisions around suitable support/help and avoidance of negative or hostile reactions*  Understands how to undertake empathic assessment in context of the Level 2 community response to distress   - 1. **Assess and respond to risk**   Be able to assess, supported by relevant tools/materials, whether the person referred is an imminent risk to themselves or others and  understand how to respond appropriately   - 1. **Providing relevant information**   Gives accurate information and guidance clearly and in a way which is appropriate to the individual |
| **Distress Brief Intervention Level 2: Delivering Distress Brief Intervention** |  |  |
| Performance objectives / competency statements | Determinant | Change objectives |
| **5.1 Engage and foster a good intervention alliance with person referred throughout the duration of the Distress Brief Intervention contact** | Knowledge & Skills  Skills  Skills  Skills & Attitudes  Skills & Attitudes  Skills  Skills | **5.1 Addressing the issue**  Recognises appropriate opportunities to discuss distress  Creates an environment conducive to discussions of mental wellbeing and distress  Raises issues around distress carefully, monitoring the impact on the person referred following distress and adapting approach as necessary  Informs on importance and relevance of discussing distress  **5.1 Interpersonal and communication skills**  Uses language which is appropriate for the person in distress and checks for understanding  Maintains appropriate levels of eye-contact and offers person in distress full attention  Adapts demeanour and tone in response to the person and topic during contacts |
| **5.2 Provide person-centred support across all relevant aspects of the Distress Brief Intervention, working *collaboratively* with the person referred following distress throughout** | Knowledge & Attitudes  Knowledge & Skills  Skills  Knowledge & Skills | **5.1, 5.2 Person-centred support**  Recognises and acknowledges the underlying determinants of distress in the context of personal histories, including trauma awareness  Identifies proximal triggers or causes of distress  Develops and records a support plan  Provides signposting and information depending on the needs of the person referred following distress |
| **5.3 Understand the importance and how to enact a compassionate response for distress** | Knowledge & Skills  Knowledge & Skills | - 1. **Compassionate response**   Understands the importance, key skills and attributes of compassionate response  *Skills: Attention; imagery; reasoning; behaviour; sensory; feeling*  *Attributes: motivation/care for wellbeing; sensitivity; sympathy; distress tolerance; empathy*  Understands how to use compassion skills and attributes in the context of an initial contact and 14-day support |
| **5.4 Use motivational interviewing techniques during Distress Brief Intervention contacts** | Knowledge  Skills  Skills  Skills  Skills | - 1. **Motivational interviewing**   Understands and acknowledges the ambivalence that the person referred following distress may feel about their actions and behaviour  Uses basic Motivational Interviewing skills (e.g. OARS) to support Distress Brief Intervention interactions  Is able to adapt to and roll with resistance during Distress Brief Intervention contacts  Provides regular feedback and summaries to structure Distress Brief Intervention **c**ontacts and transition to other components of the Distress Brief Intervention  Encourages personal responsibility |
| **5.5 Use cognitive behavioural techniques to understand distress and identify suitable strategies of support** | Knowledge  Skills  Skills | **5.5 Cognitive behavioural approach**  Understands the relevance of a cognitive behavioural approach to distress  Support a person referred following distress to explore interrelated thoughts and behaviours relevant to their distress  Uses appropriate materials or tools to support a person referred following distress to understand and address unhelpful (i) thoughts, (ii) behaviours |
| **5.6 Understands the relevance of health behaviour change and can support individuals referred following distress to change behaviours** | Knowledge & Skills  Skills  Skills | **5.6 Behaviour change methods and techniques**  Identify relevant methods and techniques of behaviour change to support the objectives of the individual referred following distress  *Example basic behaviour change techniques: goal setting (SMART: Specific, Measurable, Achievable, Relevant, Timely) to achieve desired outcomes, supportive action plans (When, Where, How) and coping plans (adaptive coping actions linked to high-risk situations) and self-monitoring*  Adapt and communicate behaviour change strategies to the needs of the individual referred following distress  Review and assess progress throughout |
| **5.7 End the period of contact as intended/by agreement, ensuring that an appropriate plan is in place for ongoing self-management or further support through signposting to other services or community resources** | Skills  Knowledge  Knowledge & Skills  Skills  Knowledge  Skills  Skills  Skills  Skills & Attitudes  Knowledge & Skills | **5.7 Self-management**  Develops and reviews support plan throughout DBI contact period,  ensuring agreed supportive actions are in place for: (i) the expected schedule of contact with DBI service (ii) maintenance or self-management beyond the schedule of Distress Brief Intervention contact  **5.7 Signposting**  Excellent knowledge and links to wide range of relevant services or support available locally and nationally  Ability to identify and access accurate information on relevant services or support locally and nationally  Directs to local services or supports as is required for those requiring further help and support  Knows or seeks clarification around eligibility, availability, accessibility and any costs prior to signposting to services  Ensures the individual is fully aware of the purpose of the signposting and understands nature of support available from the signposted service  Communicates with signposted service to refine or modify signposting actions  **5.7 Managing endings**  Uses summary and review to communicate key issues and developments throughout DBI contact  Focuses on positives and validates progress towards desired outcomes  Links *the above* to agreed actions for ongoing self-management and/or additional support beyond the DBI contact period |
| **5.8 Following assessment or contact, makes appropriate decisions over suitable referral routes for individuals who require support or help beyond the scope or remit of the Distress Brief Intervention** | Knowledge  Skills  Knowledge & Skills  Knowledge & Skills | **5.8 Risk assessment and decision making**  Identify vulnerable individuals with complex health issues which require specialist assessment and/or support  Makes use of appropriate materials and tools to support assessment and decision making  Provides accurate information materials or resources  Seeks advice or guidance on complex health issues in line with local protocols |
